# The antimicrobial, antibiofilm, and wound healing properties of ethyl acetate crude extract of an endophytic fungus *Paecilomyces* sp. (AUMC 15510) in earthworm model

**DOI:** 10.1038/s41598-022-23831-4

**Published:** 2022-11-10

**Authors:** Shimaa H. Salem, Saad S. El-Maraghy, Ahmed Y. Abdel-Mallek, Mohamed A. A. Abdel-Rahman, Emad H. M. Hassanein, Osama A. Al-Bedak, Fatma El-Zahraa A. Abd El-Aziz

**Affiliations:** 1 Fungal Physiology Laboratory, Botany and Microbiology Department, Faculty of Science, Assiut University, Assiut, Egypt; 2 Mycology Laboratory, Botany and Microbiology Department, Faculty of Science, Assiut University, Assiut, Egypt; 3 Plant Protection Research Institute, Agricultural Research Center, Dokki, Giza Egypt; 4 Department of Pharmacology and Toxicology, Faculty of Pharmacy, Al-Azhar University-Assiut Branch, Assiut, Egypt; 5 Assiut University Mycological Centre (AUMC), Assiut University, Assiut, Egypt; 6 Department of Zoology, Faculty of Science, Assiut University, Assiut, 71516 Egypt

**Keywords:** Microbiology, Zoology

## Abstract

The endophytic fungus *Paecilomyces* sp. (AUMC 15510) was isolated from healthy stem samples of the Egyptian medicinal plant *Cornulaca monacantha*. We used GC–MS and HPLC analysis to identify the bioactive constituents of ethyl acetate crude extract of *Paecilomyces* sp. (PsEAE). Six human microbial pathogens have been selected to evaluate the antimicrobial activity of PsEAE. Our data showed that the extract has significant antimicrobial activity against all tested pathogens. However, the best inhibitory effect was observed against *Bacillus subtilis* ATCC 6633 and *Pseudomonas aeruginosa* ATCC 90274 with a minimum inhibitory concentration (MIC) of 3.9 μg/ml and minimum bactericidal concentration (MBC) of 15.6 μg/ml, for both pathogens. Also, PsEAE exerts a significant inhibition on the biofilm formation of the previously mentioned pathogenic strains. In addition, we evaluated the wound healing efficiency of PsEAE on earthworms (*Lumbricus castaneus*) as a feasible and plausible model that mimics human skin. Interestingly, PsEAE exhibited a promising wound healing activity and enhanced wound closure. In conclusion, *Paecilomyces* sp. (AUMC 15510) could be a sustainable source of antimicrobial agents and a potential therapeutic target for wound management.

## Introduction

Recently, a wide spread of multidrug-resistant strains represents a serious threat to patients' life worldwide^1^. This issue is considered a real obstacle to the pharmaceutical industry in producing efficient drugs against multidrug-resistant pathogens^2,3^. Therefore, it is necessary to discover an alternative sustainable source of novel and promising antimicrobial agents. It is worth mentioning that there is a tight relationship between the wound healing process and microbial infection^4,5^. The wound healing process is the regeneration of the damaged tissue after injury^6^. Wound healing start with hemostasis, including vascular constriction, platelet aggregation, and fibrin formation. The inflammation cascade follows this step as a spontaneous response to the injury. Then, the proliferation stage includes re-epithelialization, angiogenesis, and collagen synthesis. Finally, the remodeling stage includes collagen remodeling and vascular maturation for tissue restoration^7–10^. This mechanism of the wound healing process is tightly regulated, and failure of this mechanism leads to the formation of chronic wounds^11,12^. Since the skin represents the primary protective barrier against all external stimuli such as microbial infection, which is the essential factor that increases the risk of non-healing chronic wounds^11,13^. There are several pathogens, such as *Pseudomonas aeruginosa* and *Staphylococcus aureus*, that retard the wound healing process through the biofilm formation that enables the aggregation of bacterial cells^14^. This reduces the antibiotic efficiency due to the difficulty of penetration into the adhesive bacterial biofilm^15^. In addition, these pathogens have virulence secretion systems that secret toxic effector proteins that recruit immune cells, increasing inflammation and prolonging healing events^16,17^. Therefore, there is an urgent need to find alternative sources of bioactive compounds rather than the available conventional antibiotics^18^. In this way, endophytic fungi represent a novel feedstock source of bioactive compounds that are widely used in various applications, including antimicrobial, antioxidant, and immunosuppressant^19,20^. The importance of endophytic fungi could be due to their ability for prolonged colonization inside the plant tissues without exerting any symptoms. This extraordinary interaction with the host plants leads to discover novel bioactive compounds that have various beneficial applications^21–23^. The unique ecological relationship of endophytic fungi with the plants acquired it with unusual biosynthetic pathways that could be the main reason for producing undiscovered secondary metabolites^19^.

Accordingly, the present study was designed to isolate endophytic fungi from wild medicinal plants and evaluate its extract's antimicrobial and wound healing activities. *Paecilomyces* sp. (AUMC 15510) was the most dominant fungal isolate; we identified the strain by sequencing the ITS region. Although *Paecilomyces* sp. extracts exert significant biological activities, there are no sufficient reports on their predicted antimicrobial and wound healing activities^24^. Therefore, this study was designed to assess for the first time the antimicrobial, antibiofilm formation, and wound healing activities of ethyl acetate crude extract of *Paecilomyces* sp. (PsEAE). We used GC–MS and HPLC analysis to determine the composition of PsEAE. Then, we evaluate the antimicrobial activity of PsEAE on four pathogenic bacterial strains that can form biofilm and two pathogenic fungi. Also, its biofilm inhibition activity was assessed as well. In addition, we used earthworms (*Lumbricus castaneus*) as a simple, feasible, and reproducible model for wound healing assessment. The earthworm has a similar triene and tetraene as compared to human skin. It has been used previously as a successful model to assess the wound healing efficacy of some nanoformulations^25–27^. We used histological examination, scanning electron microscopy ^28^, and transmission electron microscopy (TEM) to evaluate the healing properties of PsEAE on the induced wounds in the tissues. The earthworm model has been used as an alternative model to higher laboratory animals for preclinic surgical studies^29^. In addition, earthworm contains photosensitive proteins similar to those found in human skin. Therefore, it has been used as a model to examine the phototoxic effects of solar UV radiation^30,31^.

## Results and discussion

### Isolation and identification of *Paecilomyces* sp. (AUMC 15510)

**Figure 1:**
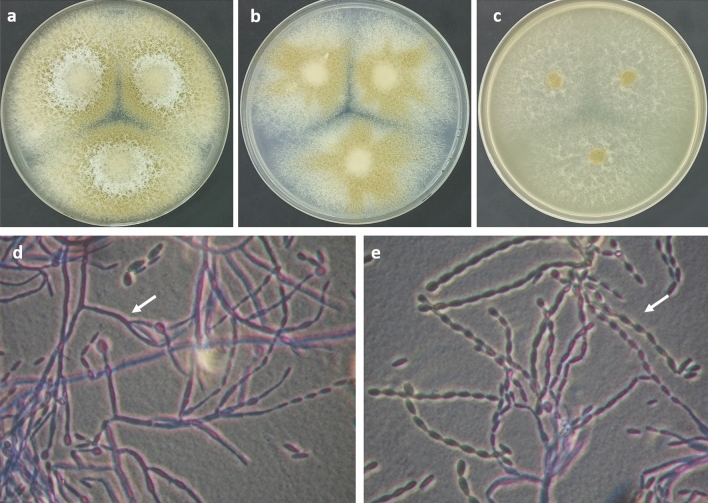
*Paecilomyces* sp. AUMC 15510: (**a**–**c**) 7-day-old colonies on PDA, Cz, and MEA at 25 °C. (**d**) Irregularly branched conidiophores with phialides. (**e**) Chains of ellipsoidal and/or cylindrical, truncate conidia. (White arrows).

The endophytic fungus *Paecilomyces* sp. (AUMC 15510) was isolated from stem samples of the medicinal plant *C. monacantha* with a colonization frequency of 80%. The fungus was identified using morphological and molecular approaches. For morphological identification, we used three types of media [Potato Dextrose Agar (PDA), Czapek's agar (CZA), and Malt Extract Agar (MEA)] to study the macroscopic and microscopic characteristics features of the colony, such as mycelium color, colony texture, conidia, and conidiophore morphology as shown in (Fig. 1). To identify the taxonomic status of the strain in relation to other members of *Paecilomyces* and *Byssochlamys*, phylogenetic analysis of the ITS dataset was used. There were 20 sequences in the total ITS collection. A total of 523 characters made up the maximum parsimony dataset, of which 445 could be accurately aligned (with no gaps or N), 220 were considered as variable characters that were parsimony-uninformative, and 32 were counted as parsimony-informative. The ideal model for nucleotide substitution was Tamura's three-parameter formulation employing a discrete Gamma distribution (T92 + G). In the dataset for maximum parsimony, 8 trees totaling 391 steps were produced with a final ML optimization likelihood value of -2087.63, consistency index of 0.767857, retention index of 0.856354, and composite index of 0.657557, the best-scoring ML tree out of the eight most parsimonious trees is shown in (Fig. S1). *Paecilomyces* sp. (AUMC 15510) was differentiated by one long distinct branch in the ITS tree. As a result, it is presented here as a potentially new species since more gene sequencing, such as β-tubulin and Calmodulin genes, is needed for precise identification. Sequences of ITS and LSU of *Paecilomyces* sp. AUMC 15510 were deposited to GenBank as OP429630 and ON685324, respectively. We isolated *Paecilomyces* sp. (AUMC 15510) as a fungal endophyte in *C. monacantha* for the first time in Egypt. However, this fungus had been isolated previously from leaves' tissues of *Edgeworthia chrysantha* (a traditional Chinese medicinal plant)^32^ and is also reported as a marine-derived fungus isolated from different coral reefs in the Red Sea in Egypt^33^.

### Gas chromatography‑mass spectrometry (GC–MS) and high-performance liquid spectrometry (HPLC) analysis

The characterization of bioactive compounds in the ethyl acetate crude extract of *Paecilomyces* sp. (AUMC 15510) was investigated by GC–MS and HPLC. GC–MS chromatogram revealed the presence of 19 peaks related to the bioactive compounds which were identified by comparing their mass spectra with those of Wiley 275 and NIST 02 library (Fig. S2). The retention time, peak area, and molecular formula of the identified compounds are presented in Table 1. The chemical compounds in the ethyl acetate crude extract of *Paecilomyces* sp. (AUMC 15510) were found to be hexanoic acid, 2-ethyl-, 2(3H)-naphthalenone,4,4a,5,6,7,8-hexahydro-1-methoxy-, 1-oxaspiro [3.5] nonan-2-one, 3-methylene-, 7-tetradecene, 4-chloro-3,5-dimethylphenol (chloroxylenol), 2,4-di-tert-butylphenol, cetene, 2,6,10-trimethyltetradecane, pentacosane, 1-eicosanol, 1-chlorooctadecane, hexadecanoic acid, methyl ester, 1-docosene, 9,12-octadecadienoic acid (Z,Z)-, methyl ester, hexadecanoic acid, 2,3-dihydroxypropyl ester, 9-octadecenoic acid, methyl ester, cis-vaccenic acid, erucic acid, and diisooctyl phthalate. The identification of these compounds has emphasized the ability of *Paecilomyces* sp. (AUMC 15510) to produce bioactive metabolites. The most important compounds identified in PsEAE were diisooctyl phthalate (DIOP) and 4-chloro-3,5-dimethylphenol (chloroxylenol) with a retention time of (29.84 min, 11.67 min) and peak area (33.77%, 33.37%) respectively. DOIP has wound healing and antimicrobial activities, as previously reported^34–36^. Moreover, chloroxylenol has antimicrobial activity and is used for skin and wound disinfection^37^. We detected various fatty acids and fatty acids esters in the ethyl acetate crude extract, such as 9-octadecenoic acid, methyl ester, hexadecanoic acid, methyl ester, 9,12-octadecadienoic acid (Z, Z)-, methyl ester, cis-vaccenic acid, and erucic acid. These fatty acids have a key role in accelerating wound healing^38,39^ and have antimicrobial properties^40–42^. HPLC analysis of PsEAE revealed the presence of different phenolic and flavonoid compounds that were identified as gallic acid, chlorogenic acid, catechin, methyl gallate, caffeic acid, pyro catechol, rutin, ellagic acid, vanillin, naringenin, daidzein, quercetin, cinnamic acid, apigenin, kaempferol, and hesperetin (Fig. S3). Table 2 showed that *Paecilomyces* sp. (AUMC 15510) produced high amounts of daidzein at 81,286.11 µg/g followed by naringenin with concentration of 27,378.15 µg/g, while caffeic acid and quercetin were detected at concentration of (7948.79 and 3652.43 µg/g), respectively. Based on the previous literatures, phenolic and flavonoid compounds have potential biological activity with different mechanisms for example daidzein has potent antioxidant, anti-inflammatory, and wound healing properties^43–46^. Also, naringenin represents a potent antioxidant molecule through it is capability of UV absorption so it has antigenotoxic and photoprotector properties^47,48^. In addition to its antioxidant capacity, naringenin has anti-inflammatory and antimicrobial activities^49^. Moreover, caffeic acid is a polyphenol that has several biological effects such as antioxidant activity^50^, anti-inflammatory activity^51^, and wound healing activity^52^. Quercetin exhibits several biological activities and potential pharmacological applications such as antioxidant^53,54^, antimicrobial^55^, anti-inflammatory^54^, and wound healing^56^. The biological activities of some compounds extracted from *Paecilomyces* sp. (AUMC 15510) were summarized in Table 3.

**Table 1:** Chemical constituents and their retention time (min) identified in the ethyl acetate crude extract of *Paecilomyces* sp. (AUMC 15510) using gas chromatography-mass spectrometry. Sl. No. serial number.

Sl. No	RT (min)	Compound name	Molecular formula	Molecular weight	Peak area %	Compound nature
1	7.90	2-Ethyl-hexanoic acid	C_8_H_16_O_2_	144	8.48	Branched chain fatty acid
2	10.37	4,4a,5,6,7,8-Hexahydro-1-methoxy-2(3H)-naphthalenone	C_11_H_16_O_2_	180	5.83	Naphthalenone derivative
3	10.51	1-Oxaspiro [3.5] nonan-2-one, 3-methylene-	C_9_H_12_O_2_	152	0.50	Methylene derivative
4	10.71	7-Tetradecene	C_14_H_28_	196	0.38	Unsaturated aliphatic hydrocarbons
5	11.67	4-Chloro-3,5-dimethyl phenol	C_8_H_9_ClO	156	33.37	Phenolic compound
6	13.56	2,4-Di-tert-butylphenol	C_14_H_22_O	206	0.33	Phenolic compound
7	14.74	Cetene	C_16_H_32_	224	1.88	Alkene
8	14.85	2,6,10-Trimethyltetradecane	C_17_H_36_	240	0.46	Alkane
9	16.69	Pentacosane	C_25_H_52_	352	0.18	Alkane
10	18.38	1-Eicosanol	C_20_H_42_O	298	1.91	Long chain fatty alcohol
11	18.48	1-Chlorooctadecane	C_18_H_37_Cl	288	0.28	Alkane
12	20.65	Hexadecanoic acid, methyl ester	C_17_H_34_O_2_	270	1.04	Fatty acid methyl ester (palmitic acid methyl ester)
13	21.70	1-Docosene	C_22_H_44_	308	1.60	Alkene
14	21.78	Hexadecanoic acid, 2,3 dihydroxypropyl ester	C_19_H_38_O_4_	330	0.70	Fatty acid propyl ester (glyceryl palmitate)
15	23.31	9,12-Octadecadienoic acid (Z,Z)-, methyl ester	C_19_H_34_O_2_	294	4.41	Fatty acid methyl ester (linoleic acid, methyl ester)
16	23.42	9-Octadecenoic acid, methyl ester, (E)-	C_19_H_36_O_2_	296	3.53	Fatty acid methyl ester (oleic acid methyl ester)
17	24.38	cis-Vaccenic acid	C_18_H_34_O_2_	282	0.52	Fatty acid (isomer of oleic acid)
18	24.73	Erucic acid	C_22_H_42_O_2_	338	0.84	*cis*13-monounsaturated fatty acid
19	29.84	Diisooctyl phthalate	C_24_H_38_O_4_	390	33.77	Phthalic acid ester

**Table 2:** HPLC analysis of phenolics and flavonoids in the ethyl acetate crude extract of *Paecilomyces* sp. (AUMC 15,510).

Peak #	RT (min)	Compound name	Molecular formula	Molecular weight	Area	Area (%)	Conc. (µg/g)
1	3.413	Gallic acid	C_7_H_6_O_5_	170.12	41.50608	0.1110	146.54
2	4.300	Chlorogenic acid	C_16_H_18_O_9_	354.31	76.94178	0.2058	452.86
3	4.701	Catechin	C_15_H_14_O_6_	290.27	6.92096	0.0185	71.62
4	5.829	Methyl gallate	C_8_H_8_O_5_	184.15	56.83606	0.1520	152.48
5	6.119	Caffeic acid	C_9_H_8_O_4_	180.16	2546.57690	6.8125	7948.79
6	6.784	Pyro catechol	C_6_H_6_O_2_	110.11	26.59203	0.0711	86.50
7	7.679	Rutin	C_27_H_30_O_16_	610.5	5.75431	0.0154	32.90
8	8.995	Ellagic acid	C_14_H_6_O_8_	302.19	63.90355	0.1710	1984.02
9	9.816	Vanillin	C_8_H_8_O_3_	152.15	19.54030	0.0523	30.12
10	10.731	Naringenin	C_15_H_12_O_5_	272.25	5822.26172	15.5755	27,378.15
11	12.130	Daidzein	C_15_H_10_O_4_	254.24	2.78816e4	74.5878	81,286.11
12	12.599	Quercetin	C_15_H_10_O_7_	302.23	682.53961	1.8259	3652.43
13	13.892	Cinnamic acid	C_9_H_8_O_2_	148.16	33.03144	0.0884	29.92
14	14.477	Apigenin	C_15_H_10_O_5_	270.24	21.44422	0.0574	73.43
15	14.913	Kaempferol	C_15_H_10_O_6_	286.24	17.23908	0.0461	84.41
16	15.666	Hesperetin	C_16_H_14_O_6_	302.28	78.19218	0.2092	193.58

**Table 3:** Biological activities of some compounds identified from ethyl acetate crude extract of *Paecilomyces* sp. (AUMC 15510).

Compounds	Biological activities	Refs.
4-Chloro-3,5-dimethylphenol (chloroxylenol)	Antimicrobial, skin and wound disinfection	^37^
Cetene	Antimicrobial and antioxidant	^57^
Pentacosane	Antioxidant and antimicrobial	^58^
1-Eicosanol	Antibacterial and antioxidant	^28,59^
Hexadecanoic acid, methyl ester	Antibacterial, antifungal, and anti-inflammatory	^60–62^
9,12-Octadecadienoic acid (Z,Z)-, methyl ester	Anti-inflammatory, antibacterial, skin repair and wound healing	^40,63,64^
9-Octadecenoic acid, methyl ester, (E)-	Anti-inflammatory, antibacterial, skin repair and wound healing	^40,63,64^
cis-Vaccenic acid	Wound healing, antibacterial, hypolipidemic and antioxidant	^39,65^
Erucic acid	Antibacterial and wound healing	^38,41^
Diisooctyl phthalate	Wound healing and antimicrobial	^34–36^
Daidzein	Antimicrobial, antioxidant, anti-inflammatory, and wound healing	^43–46^
Naringenin	Photoprotective and antigenotoxic properties, antioxidant, anti-inflammatory, and antimicrobial	^47–49^
Caffeic acid	Antioxidant, anti-inflammatory, antibacterial, anticarcinogenic, and wound healing	^50–52^
Quercetin	Antioxidant, anti-inflammatory, anticancer, antimicrobial, and wound healing	^53–56^

### Antimicrobial activity of PsEAE

**Figure 2:**
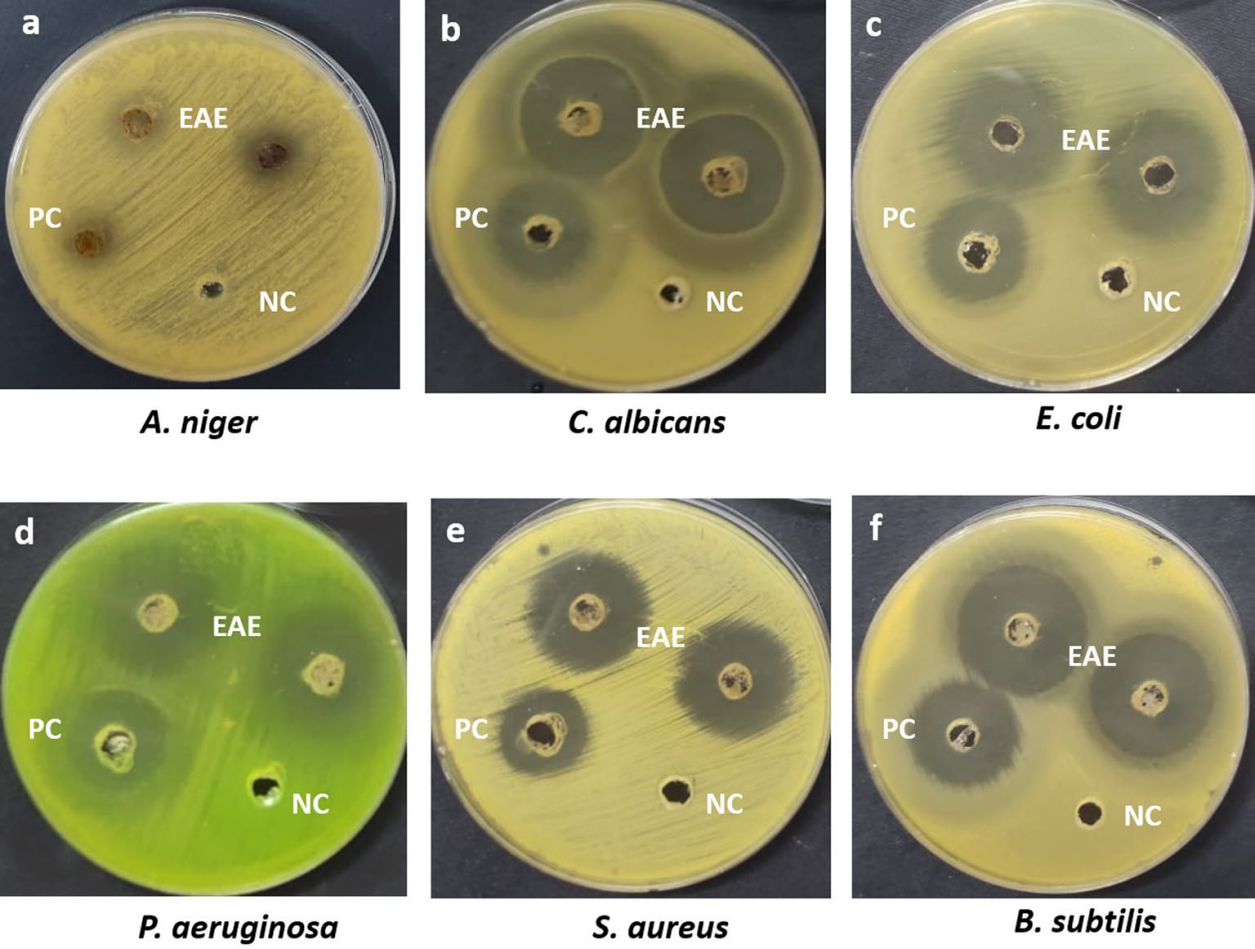
The inhibition zone (mm) of ethyl acetate crude extract (EAE) of *Paecilomyces* sp. (AUMC-15510) at a concentration of 5 mg/ml against (**a**) *A. niger* (**b**) *C. albicans* (**c**) *E. coli* (**d**) *P. aeruginosa* (**e**) *S. aureus*, and (**f**) *B. subtilis*. PC: Fluconazole and Gentamicin at concentration of 5 mg/ml (positive control); NC: 10% DMSO (negative control).

Several natural compounds' antimicrobial activity has attracted attention in the last few years, and various attempts have been made to use natural extracts to combat the different pathogenic strains^66^. The antimicrobial activity of PsEAE at a concentration of 5 mg/ml was preliminarily tested against different pathogens such as *B. subtilis* ATCC 6633, *S. aureus* ATCC 6538, *E. coli* ATCC 8739, *P. aeruginosa* ATCC 90274, *C. albicans* ATCC 10221, and *A. niger* using the agar well diffusion method. The results showed that the extract was efficiently suppressing the growth of all tested pathogens. In this experiment, we measured the zone of inhibition and the data presented in (Fig. 2, Table 4). The crude extract recorded the highest zone of inhibition against *P. aeruginosa* (31.6 ± 0.58 mm), while the lowest zone of inhibition was reported against *A. niger* (11.3 ± 0.58 mm). All the results of inhibition zones were compared with the positive control gentamicin and fluconazole.

**Table 4:** Antimicrobial activity of ethyl acetate crude extract of endophytic fungus *Paecilomyces* sp. (AUMC 15510) isolated from stem of *C. monacantha* in the agar diffusion assay.

Extract	Inhibition zone diameter (mm)					
	*B. subtilis*	*S. aureus*	*E. coli*	*P. aeruginosa*	*C. albicans*	*A. niger*
PsEAE	30.3	24.3	30.3	31.6	29.6	11.3
Gentamicin*	20.3	18.3	19.6	20.3		
Fluconazole*					21.3	8.3

PsEAE—ethyl acetate crude extract obtained from the culture filtrate of *Paecilomyces* sp. in PDB medium. Experiments were done in triplicates. Standard deviation value is ± 0.58 for all tested pathogens and control.

*Gentamicin and fluconazole were used as a positive control.

### MIC, MBC, and MFC of the PsEAE of the endophytic fungus *Paecilomyces* sp. (AUMC 15510)

The PsEAE was further evaluated for its MIC, MBC, or MFC using the microdilution assay, as shown in Table 5. PsEAE was active against all tested pathogens BS, SA, EC, PA, CA, and AN. The MIC values of PsEAE ranged from 3.9 to 31.5 μg/ml. The extract was strongly active against *B. subtilis* and *P. aeruginosa* with MIC of 3.9 μg/ml for both, followed by *E. coli*, *S. aureus*, and *C. albicans* with MIC of 7.8, 15.6, and 31.5 μg/ml, respectively. Furthermore, the MBC or MFC values of the extract ranged from 15.6 to 62.5 μg/ml, showing bactericidal or fungicidal actions (MBC/MIC ≥ 4)^67^.

**Table 5:** Minimum inhibitory (MIC), minimum bactericidal (MBC), and minimum fungicidal concentrations (MFC) of (PsEAE) metabolites from *Paecilomyces* sp. (AUMC 15510) against different pathogens.

Target pathogens	PsEAE concentration (μg/ml)	
	MIC	MBC or MFC
*B. subtilis*	3.9	15.6
*S. aureus*	15.6	31.25
*E. coli*	7.8	15.6
*P. aeruginosa*	3.9	15.6
*C. albicans*	31.5	62.5
*A. niger*	31.5	62.5

PsEAE—ethyl acetate crude extract of *Paecilomyces* sp.

### Qualitative and quantitative assessment of biofilm formation

**Figure 3:**
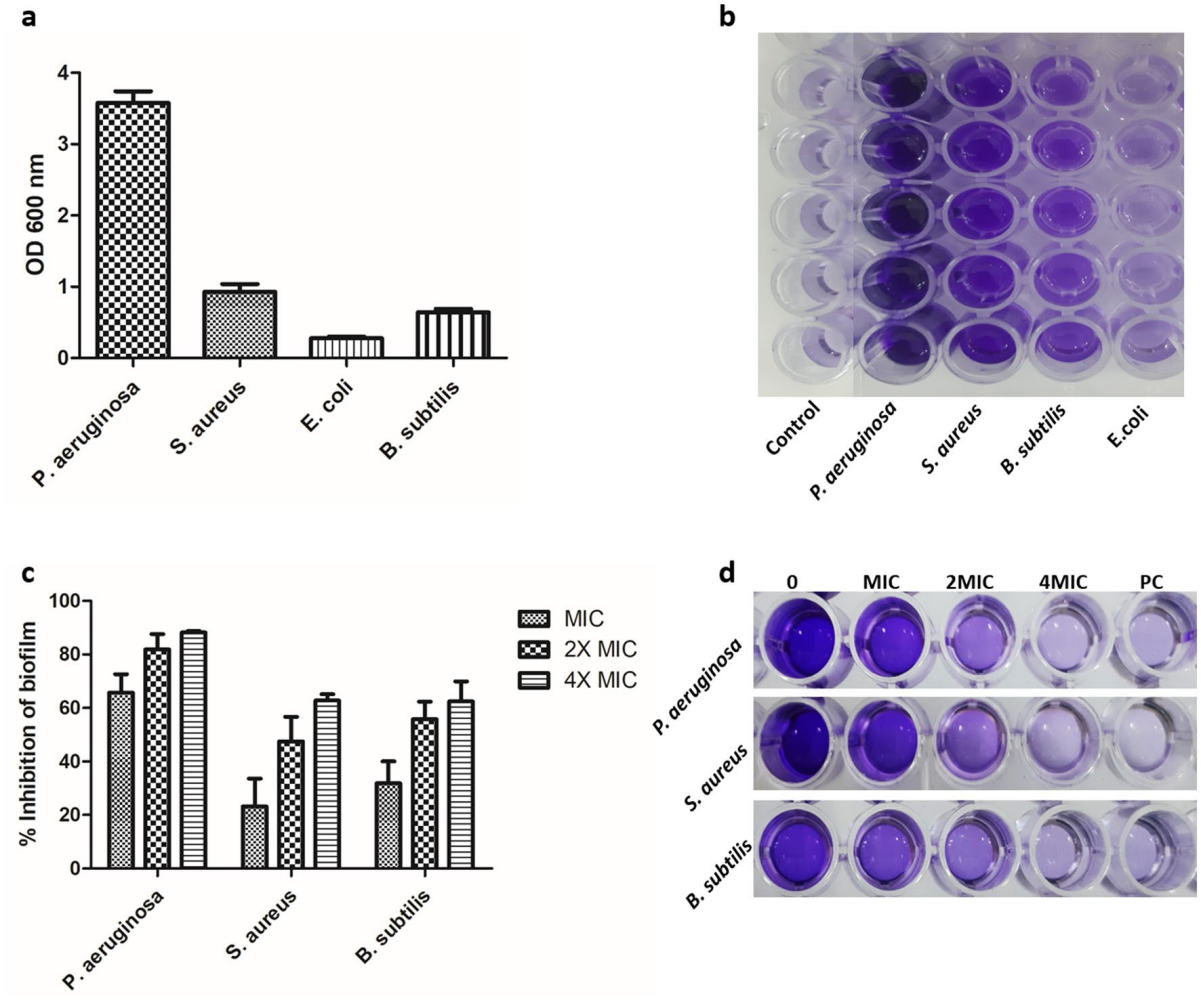
(**a**) Biofilm biomass assessment by crystal violet staining (OD 600 nm) of four bacterial strains *P. aeruginosa*, *S. aureus*, *E. coli*, and *B. subtilis* after 48 h in Brain Heart Infusion broth (BHIB) supplemented with 2% glucose. (**b**) The 96-well microtiter plate assay showed biofilm formation by four bacterial strains. (**c**) Antibiofilm efficacy of ethyl acetate crud extract from *Paecilomyces* sp. (AUMC 15510) on *P. aeruginosa*, *S. aureus*, and *B. subtilis* as assessed by crystal violet quantification of biofilm. (**d**) The 96-well microtiter plate assay depicted the effect of ethyl acetate crud extract on the biofilm formation of *P. aeruginosa*, *S. aureus*, and *B. subtilis*. PC: Gentamicin (Positive Control). The error bars on the graph represent standard deviations as a percentage of biofilm inhibition.

The biofilm production ability of four bacterial strains *B. subtilis*, *S. aureus*, *E. coli*, and *P. aeruginosa* was qualitatively evaluated using CRA assay and tube staining assay. After this screening for biofilm formation, all bacterial strains showed the ability to produce biofilm. In CRA assay, the tested strains grew as black-colored colonies, and this result confirmed that the strains have the ability of biofilm formation (Fig. S4a). To examine the thickness of biofilm, the tube staining method was used, and our results revealed that only one strain (*E. coli*) was weak for biofilm production (Fig. S4b). The quantification of biofilm produced by *P. aeruginosa*, *S. aureus*, *B. subtilis*, and *E. coli* was performed using a microtiter plate assay (Fig. 3b). The data were expressed in terms of the average OD values at 600 nm. Figure 3a shows that all tested bacterial strains could produce biofilm with different amounts. Based on the OD values of biofilm, the strains were classified as weak (*E. coli*), moderate (*S. aureus*, *B. subtilis*), and strong (*P. aeruginosa*) biofilm producers, as described by Stepanović et al.^68^.

### Effect of *Paecilomyces* crude extract on biofilm formation

Bacterial biofilms play a critical role in the delay of the wound healing process through the aggregation of bacterial cells^14^. This mode of bacterial growth is associated with 65–80% of all clinical infections and leads to higher levels of conventional antibiotic resistance^69^. Recently, Cheng et al. used antimicrobial peptides encapsulated into PLGA microspheres to inhibit the biofilm formation of pathogens isolated in the infected bone, significantly enhancing the healing of the fracture^70^. Therefore, there is an urgent need to develop and search for new therapeutic agents rather than the available conventional antibiotics. In this respect, using a microtiter plate assay, the ethyl acetate crude extract was evaluated for its potential to inhibit biofilm formation by *P. aeruginosa*, *S. aureus*, and *B. subtilis* (Fig. 3d).

Based on the percentage of biofilm inhibition, the crude extract exhibited significant antibiofilm activity against all tested bacterial pathogens (Fig. 3c). In this experiment, bacterial biofilms were exposed to multiple MIC concentrations (MIC, 2MIC, and 4MIC values) of the extract for 48 h. For *P. aeruginosa*, *S. aureus*, and *B. subtilis*, the crude extract showed the highest antibiofilm activity at 4 MIC with inhibition percentages of 88.2%, 62.8%, and 62.46%, respectively (Fig. 3c).

### In vivo wound healing effect of PsEAE in earthworm model

**Figure 4:**
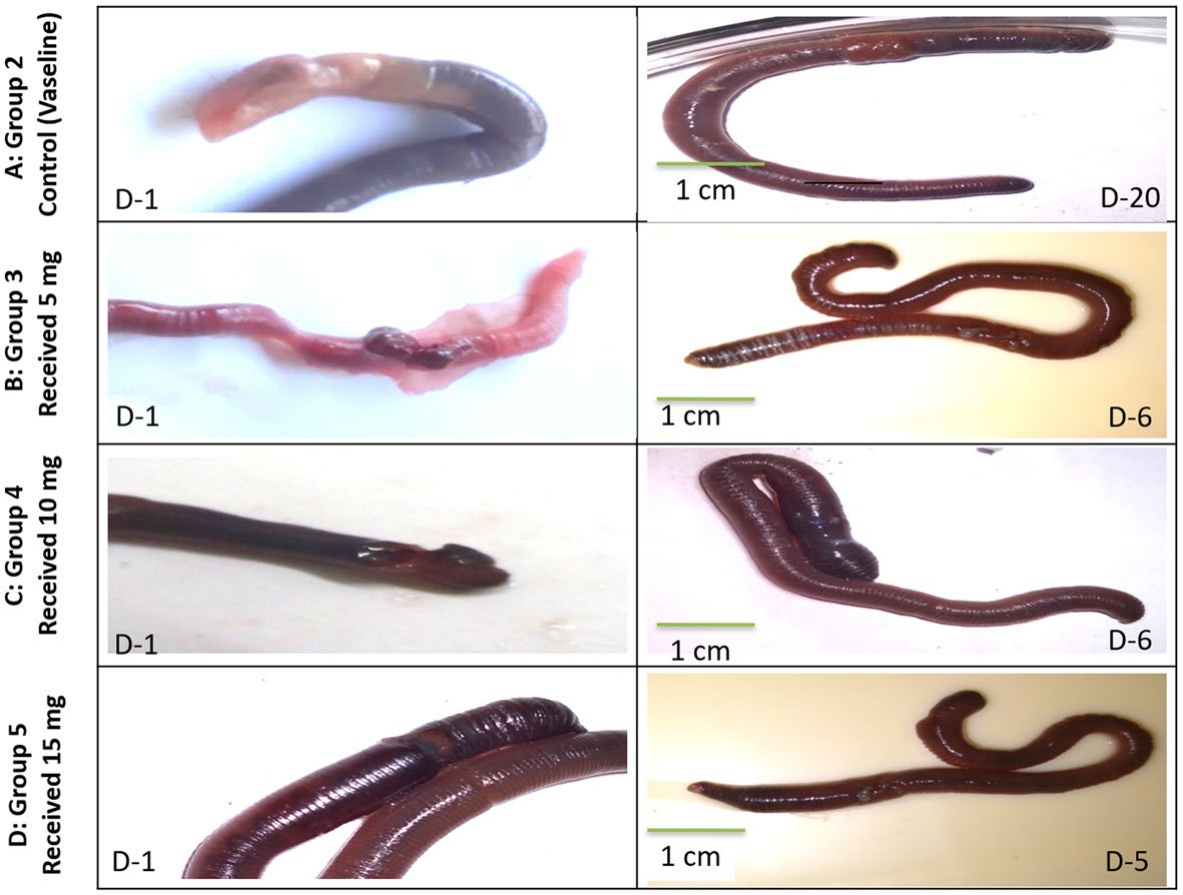
Macroscopic observation of the different groups of earthworms (*Lumbricus castaneus*) after induction of surgical wounds and examination of wound healing; (**a**) worms received Vaseline; (**b**) worms received 5 mg; (**c**) worms received 10 mg, and (**d**) worms received 15 mg.

As we mentioned previously, earthworms are a successful model for assessing wound healing properties of bioactive compounds because their structure mimics human skin features^25–27^. There is no mortality observed during the experiment up to 20 days. All the conserved symptoms of the inflammation have been observed on the first day of the induced injury, including redness, hemorrhage, edema, and exudation around the wound region. Interestingly, Group 5 showed a significant and fast wound healing process after 5 days only of the treatment with the PsEAE. Besides, groups 3 and 4 showed enhanced wound healing after six days of treatment with the PsEAE. However, group 2 that received Vaseline only as a vehicle exhibited improvement in the wound healing process after 20 days (Fig. 4). Similar observations have been recorded for wound healing in earthworm *Eudrilus eugeniae* that took 24 days to mend its posterior section in another investigation^71^. These results clearly showed the promising healing properties of PsEAE that could be attributed to the bioactive compounds that accelerate wound healing, as shown in (Table 3). Also, the potent antimicrobial activity of PsEAE prevents wound infection. Several reports showed different endophytic fungi extracts' extraordinary wound healing properties^72,73^. Recent study has shown a remarkable wound healing properties of natural extracts of *Rosmarinus officinalis* L. to emphasize the importance of discovering novel and safe bioactive compounds from natural sources^74^.

### Histological observation

**Figure 5:**
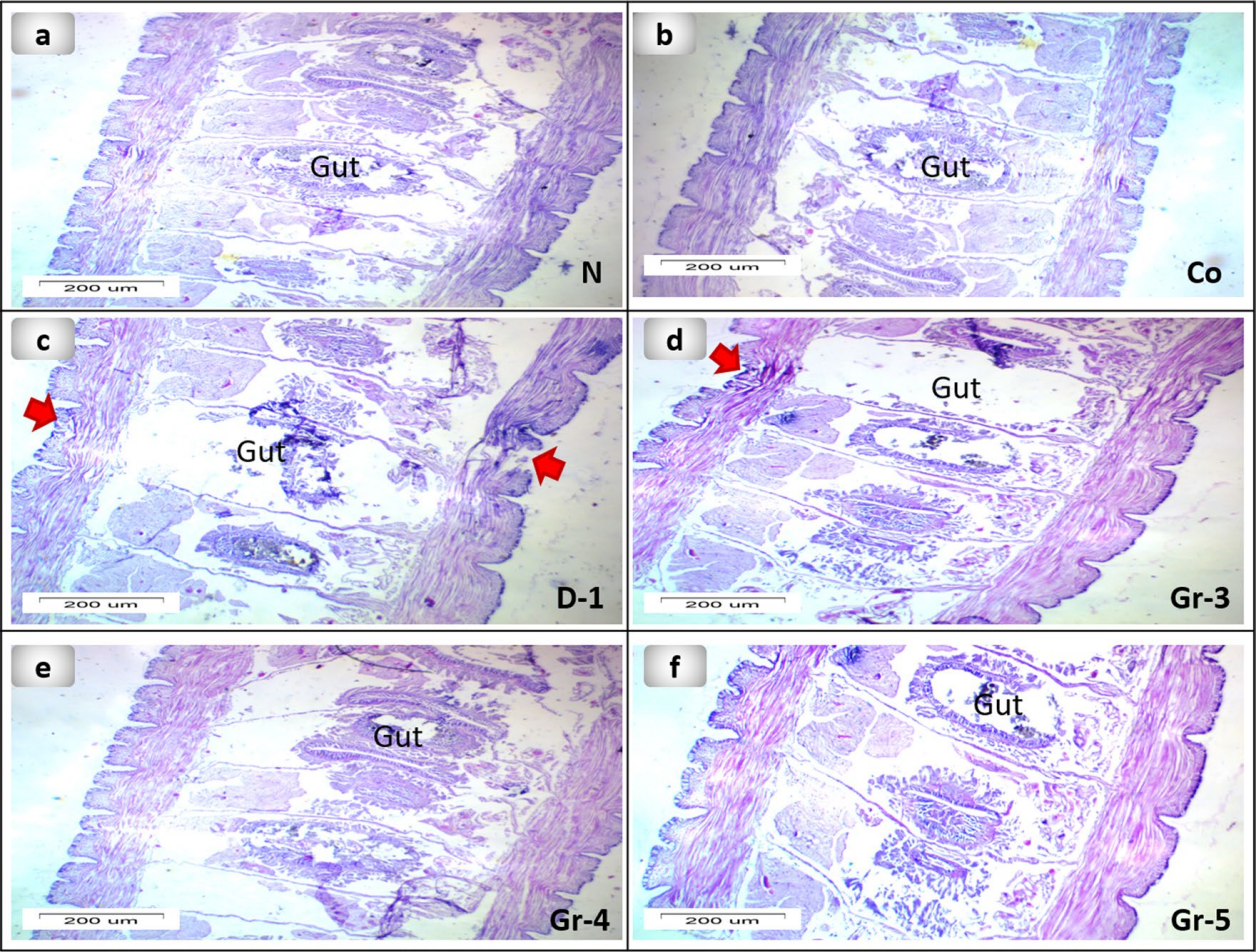
Photomicrographs of the longitudinal section of the different groups of earthworms (*Lumbricus castaneus*) after induction of surgical longitudinal wounds and examination of wound healing; (**a**) normal earthworm; (**b**) worms received Vaseline; (**c**) worm on the first day of injury, and (**d**) worms received 5 mg, (**e**) worms received 10 mg, (**f**) worms received 15 mg, note: the cells are loosely packed at the amputated region (raw). Hematoxylin and Eosin (H&E).

**Figure 6:**
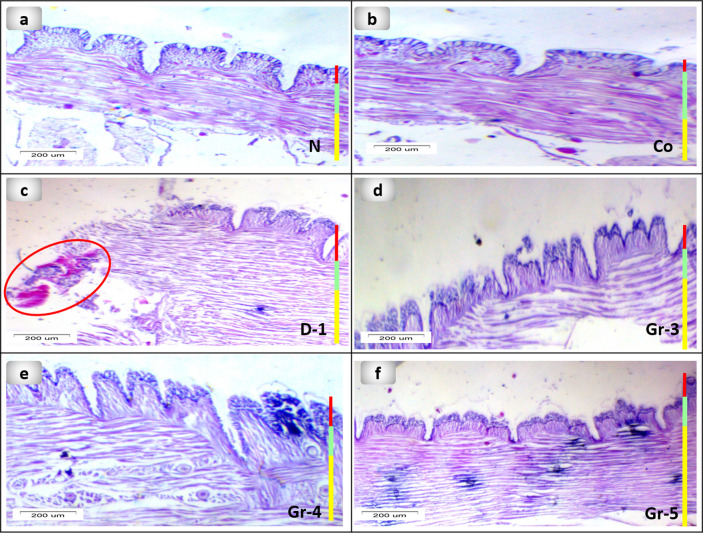
Photomicrographs of longitudinal section of the different groups of the body wall of earthworms (*Lumbricus castaneus*) after induction of surgical longitudinal wounds and examination of wound healing; (**a**) normal earthworm; (**b**) worms received Vaseline; (**c**) worm in the first day of injured showing hemorrhage (circle), and (**d**) worms received 5 mg, (**e**) worms received 10 mg, (**f**) worms received 15 mg. (Red (glandular epithelial cell layer), green (circular muscle layer) and yellow (longitudinal cell layer) Hematoxylin and eosin (H&E).

Histological assessment was used to examine the structure of the earthworm's skin. (Fig. 5a) showed the longitudinal section of the earthworm's normal structure composed of cuticle, epidermis, and circular and longitudinal muscles. The annelid epidermis is a monolayered epithelium that includes glandular, ciliated, behind, and sensory cells encased in a collagen fiber-based cuticle^75^. Figures 5 and 6 showed the comparison between the PsEAE -treated groups 3, 4, and 5 and the untreated group 2. A longitudinal section of group 2 of worms exhibits circular degeneration with a considerable infiltration of inflammatory cells and longitudinal muscles, as well as vacuolization and cell hypertrophy. In the body wall of groups 3 and 4 of worms, after 6 days of the treatment, inflammatory cells disappeared, and epidermal circular, longitudinal muscle layer restored its natural structure partly as promising signs for the enhanced wound healing process as illustrated in the longitudinal section of earthworm. However, some fractures show incomplete recovery for groups 3 and 4. Interestingly, group 5 restored the normal structure of the tissue as a sign of complete healing of wounds within five days only. The enhancement at the tissue structure level could be due to the ability of PsEAE to improve cell adhesion properties. Also, group 5 exhibited natural crawling activity as a sign of reciprocal contraction of the circular and longitudinal muscles that agrees with the description of normal earthworm peristalsis^76^.

### Scanning electron microscopy observation

**Figure 7:**
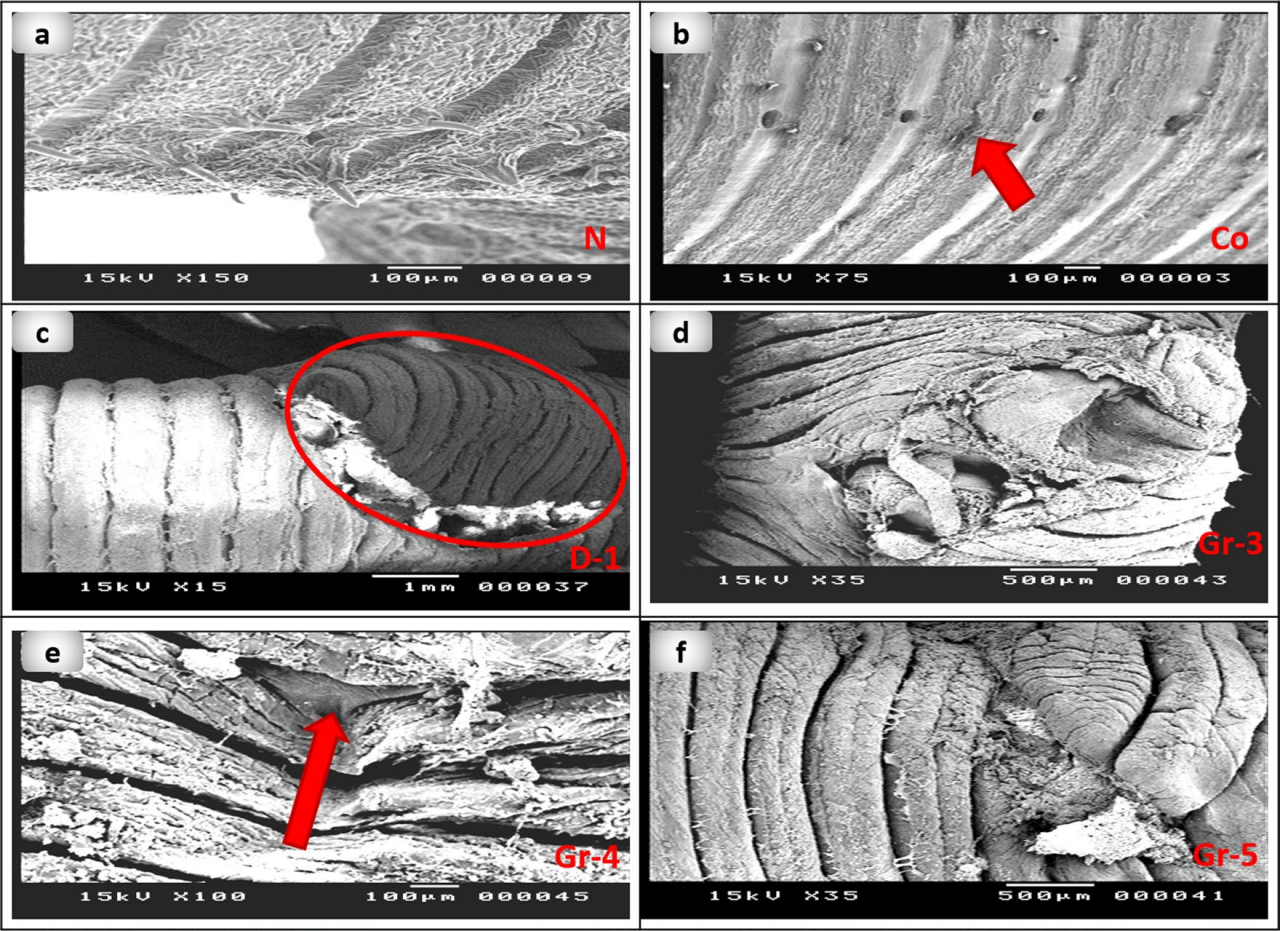
Scanning electron photomicrograph of the anterior part of earthworms (*Lumbricus castaneus*) after induction of surgical wounds and examination of wound healing; (**a**) normal earthworm; (**b**) worms received Vaseline, showing fissure(raw); (**c**) worm in first day of injured showing the coelomic fluid emerged as well as the blood surrounds the wound appearance (circle), and (**d**) worms received 5 mg, (**e**) worms received 10 mg showing fissure(raw), (**f**) worms received 15 mg.

SEM was used to examine the wound surface and to assess the regenerating epidermis surface at the wound surface after treatment with PsEAE. The wounds' surfaces in group 5 were completely closed after five days of applying PsEAE, the regenerated epidermis looked like control, and an obvious crust layer appeared. The wound surface in groups 3 and 4 is almost closed after six days. However, the crust layer formation is not formed completely (Fig. 7).

### Semithin sections observation

**Figure 8:**
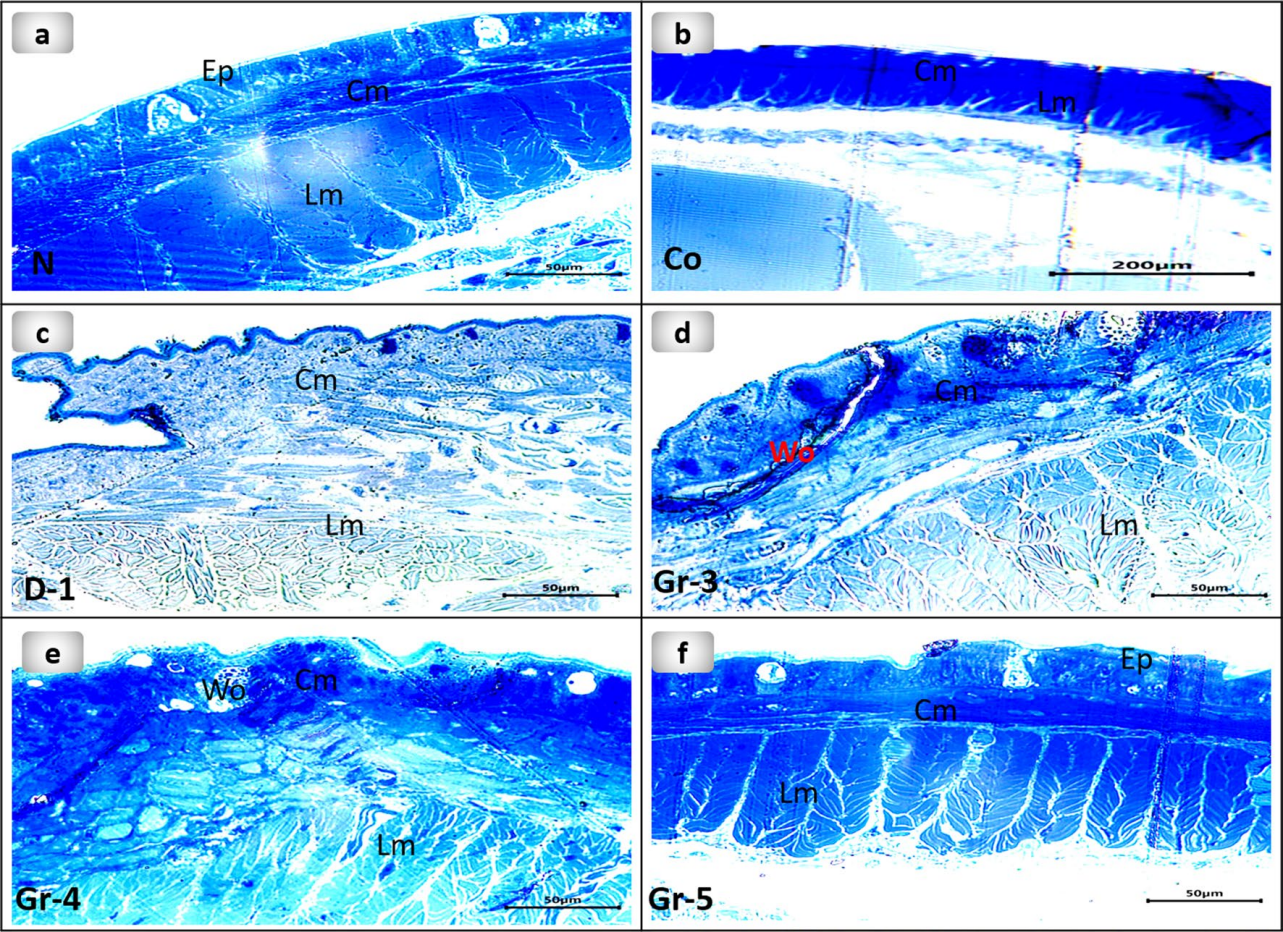
Photomicrographs of semithin sections of the earthworms (*Lumbricus castaneus*) (**a**) normal earthworm; (**b**) worms received Vaseline, showing fissure(raw); (**c**) worm on the first day of injured showing the coelomic fluid emerged as well as the blood surrounds the wound appearance (circle), and (**d**) worms received 5 mg, (**e**) worms received 10 mg showing fissure(raw), (**f**) worms received 15 mg. *Cm* Circular muscle, *Ep* Epidermis, *Lm* Longitudinal muscle, *Wo* Wound.

Normal group 1 earthworms' photomicrograph of semithin sections showed normal structural features and intact structure of cuticle, and epidermis, followed by the circular and longitudinal muscles. The untreated group 2 that received Vaseline only showed normal structure after 20 days. Although groups 3 and 4 showed wound closure after six days, the semithin study exhibited structural loss and exposed a leaning to develop excess glandular epithelium with the disintegration of the cuticular membrane, ectodermal layer, and development of spaces between the longitudinal muscles. Semithin sections of group 5 showed vanishment of the wound and inflammatory cells. It is worthy to mention that the skin of earthworms that received 15 mg of PsEAE promoted wound healing on the fifth day with the complete structure of epidermal, circular, and longitudinal muscles (Fig. 8).

### Transmission electron microscopy (TEM) observation

**Figure 9:**
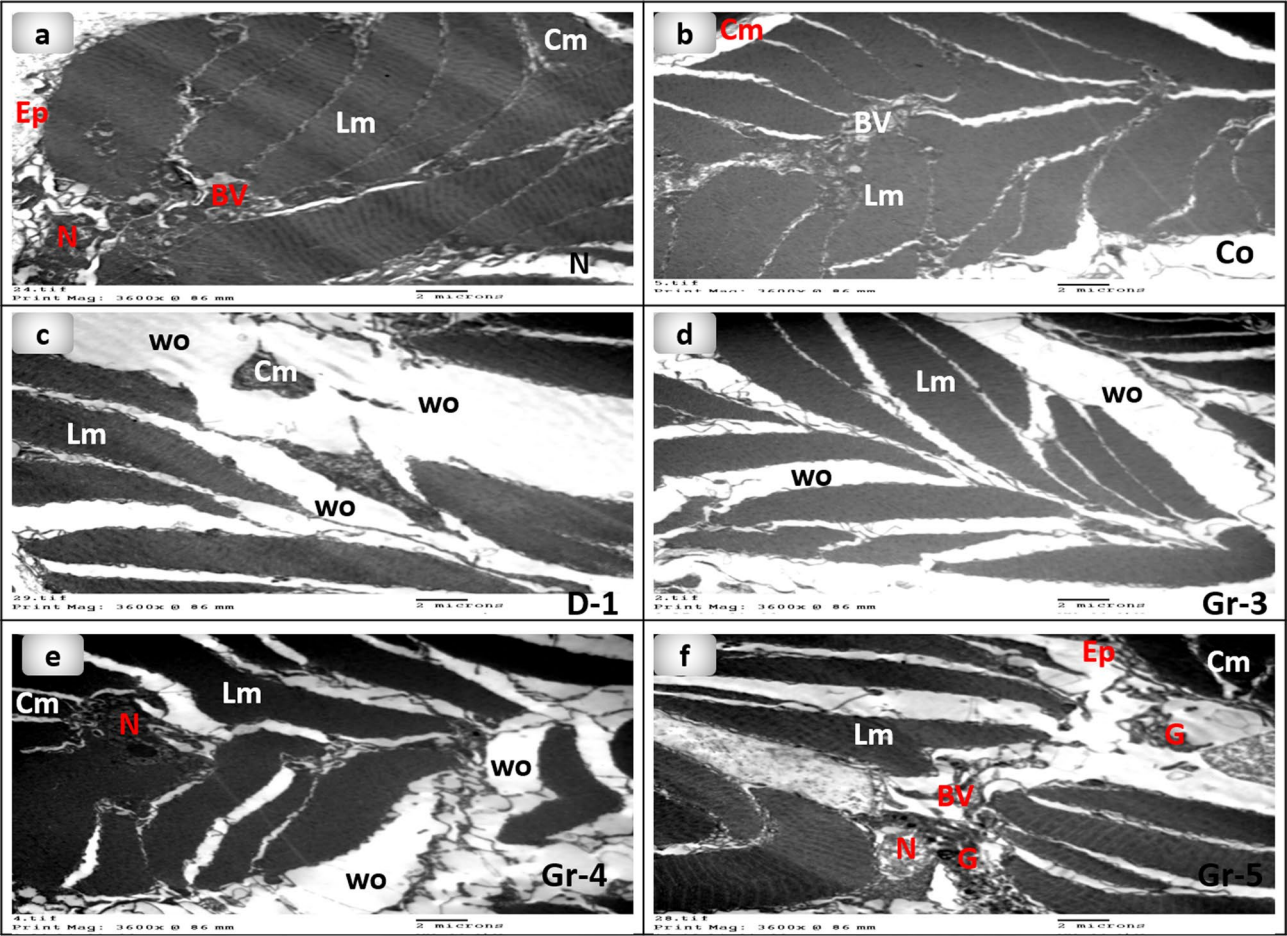
Transmission electron microscopy micrographs of earthworms (*Lumbricus castaneus*) (**a**) normal earthworm; (**b**) worms received Vaseline, showing fissure (raw); (**c**) worm on the first day of injured showing the coelomic fluid emerged as well as the blood surrounds the wound appearance (circle), and (**d**) worms received 5 mg, (**e**) worms received 10 mg showing fissure(raw), (**f**) worms received 15 mg. *Cm* Circular muscle, *Ep* Epidermis, *BV* Blood vessel, *G* Granules, *Lm* Longitudinal muscle, *N* Nucleus, *Wo* Wound.

The skin's TEM micrographs of control group 1 and the untreated group 2 are shown in (Fig. 9a,b). Some damage features were still observable in groups 3 and 4, including cuticle establishment, epidermis degradation, and necrotic circular muscles. Also, the intercellular matrix was loose and edematous, allowing for minute vessel extension and the generation of new capillaries. In addition, fibroblast proliferation was observed. Conversely, the skin of earthworms in group 5 that were treated with 15 mg of PsEAE showed an almost similar structure compared to the control group. Granulation tissue was formed due to the development of fibroblasts, capillaries, and collagen in response to the wound (Fig. 9). Similar observations were recorded previously that showed the development of capillaries, fibroblasts, collagen, and forming granulation tissue during wound healing^77^.

## Conclusion

*Cornulaca monacantha* is a wild medicinal plant that grows widely in the desert of Egypt under highly stressed conditions of temperature, salinity, and less water availability. This harsh environment acquired the plant's natural protection to resist pests and common diseases. Therefore, this plant is a rich source of endophytic fungi that showed intricate interaction mechanisms with different pathways to secrete various secondary metabolites to enable plant growth under abiotic stress condition. Our study isolated and identified *Paecilomyces* sp. (AUMC 15510) as the dominant endophytic fungi that colonized *C. monacantha.* Then, we used ethyl acetate to extract the major bioactive compounds that were secreted by the fungi. PsEAE exhibited potent antimicrobial activity against pathogens that can form biofilm. Also, the topical application of PsEAE on the wounds conducted in earthworms showed a significant potency in wound healing. The dual function of PsEAE could be attributed to the novel bioactive compounds constituent that activates the cell migration, regeneration of the damaged tissues, and its recorded antimicrobial properties. Endophytic fungi represent a sustainable source of biologically active secondary metabolites that are considered a promising alternative to custom antibiotics for the pharmaceutical industry.

## Materials and methods

### Isolation of *Paecilomyces* sp. (AUMC 15510)

The fresh healthy samples from the leaf and stem of the medicinal plant *Cornulaca monacantha* were collected from Wadi El Assuti, Assuit governorate, Egypt, in September 2020 (Fig. S5). The plant samples were identified by Dr. Ibrahim Nafady, the director of Wadi El-Assiuti Protectorate, and the identified specimen was kept at the herbaria of the Department of Botany and Microbiology, Assiut University (ASTU). Wadi El-Assiuti Protectorate approved permission to collect the plant, and all the experimental research was conducted according to the guidelines and legislation of Wadi El-Assiuti Protectorate and Assiut University. Fifty randomly selected segments were surface sterilized (25 segments for leaf and stem samples) as described by Araújo et al.^78^. Briefly, the tissues (leaves and stems) were washed in running tap water to remove dust. After that, surface sterilization was done by immersing plant tissues in 70% ethanol for 5 min, 2% sodium hypochlorite for 5 min, and again in 70% ethanol for 30 s. Then rinsed in sterilized distilled water multiple times and dried using a sterilized paper towel. After sufficient surface sterilization, the plant tissues were cut into small segments (2 cm), inoculated on PDA plates, and incubated at 25 °C for 10–15 days. The strain was preserved and deposited in the Assiut University Mycological Centre as* Paecilomyces* sp. (AUMC 15510).

### Molecular identification and phylogenetic analysis of *Paecilomyces* sp. (AUMC 15510)

The molecular identification of the most dominant fungal isolate was done by sequencing of ITS and a large subunit of ribosomal RNA genes (LSU) to confirm the morphological identification. The PCR amplification of ITS was done using the primers ITS1 and ITS4^79^, and the sequences of LSU rDNA were amplified with LROR-LR7 primers^80^. The sequencing of ribosomal RNA genes (rDNA) was performed by SolGent Co. (Yuseong-Gu, Daejeon, South Korea). The resulting sequences were compared with available data in the NCBI database (https://www.ncbi.nlm.nih.gov/) using BLAST search. Sequences of the closely related species belonging to genus *Paecilomyces,* and *Byssochlamys,* including sequences of the available type and ex-type specimens, were obtained from GenBank and aligned with MAFFT (version 6.861b). The phylogenetic tree was generated using MEGA X version 10.2.6^81^.

### Preparation of PsEAE

For cultivation, the endophytic fungus was inoculated in 250 mL Erlenmeyer flasks containing 100 mL of Potato Dextrose Broth (PDB) medium by adding six agar-grown mycelial plugs (5 mm) from 7-days PDA plates. The flasks were incubated at 25 °C for 21 days on a rotary shaker at 150 rpm. The fungal fermentation broth was extracted three times by adding an equal volume of ethyl acetate (EtOAc) in a separating funnel. A rotary evaporator was used to evaporate the extract to dryness to obtain a crude ethyl acetate extract. The dry crude metabolites were then dissolved in DMSO at 5 mg/ml stock solutions and kept for chromatographic characterization and biological evaluation.

### GC–MS analysis of PsEAE

The identification of active secondary metabolites from the crude extract was performed using GC-TSQ 8000 mass spectrometer (Thermo Scientific, Austin, TX, USA) coupled with a direct capillary column TG–5MS with dimensions of 30 m × 0.25 mm × 0.25 µm film thickness. The initial temperature of the column oven was held at 60 °C and programmed to 250 °C at a rate of 5 °C/min, then kept constant at 300 °C for 30. The temperature of the injector was set at 270 °C. Helium was used as a carrier gas with a constant flow rate of 1.0 ml/min. 1 µl of diluted samples were injected automatically using Autosampler AS3000. EI mass spectra were collected at 70 eV ionization voltages over the range of m/z 50–650 in full scan mode. The transfer line and ion source were set at 280 °C and 250 °C, respectively. The active components' mass spectrum was interpreted using Wiley 275 and NIST 02 mass spectral database^82^.

### HPLC analysis of phenolics and flavonoids in PsEAE

The analysis of phenolic and flavonoid compounds in PsEAE was performed using HPLC (Agilent 1260 series). The separation of compounds was carried out using Eclipse C18 column (250 mm × 4.6 mm I.D; particle size 5 μm). The temperature of column was maintained at 40 °C and the injection volume was adjusted to 5 μl for each of the sample solutions. The compounds were separated using a gradient mobile phase composed of water (A) and 0.05% trifluoroacetic acid in acetonitrile (B) with a flow rate 0.9 ml/min. The mobile phase gradient profile was set as follows: 0 min (82% A); 0–5 min (80% A); 5–8 min (60% A); 8–12 min (60% A); 12–15 min (82% A); 15–16 min (82% A) and 16–20 (82%A). The detection of resolved compounds was done by using a multi-wavelength detector that was monitored at 280 nm. The identification of compounds was performed based on the available standers of phenolic and flavonoid.

### Biological evaluations of ethyl acetate crude extract

#### Antimicrobial assay

##### Microorganisms used and inoculum preparation

The antimicrobial activity of PsEAE was assessed against pathogenic gram-positive bacteria [*Bacillus subtilis* 6633 (*B. subtilis*), *Staphylococcus aureus* 6538, (*S. aureus*)], gram-negative bacteria [*Escherichia coli* 8739 (*E. coli*), *Pseudomonas aeruginosa* 90274 (*P. aeruginosa*)], as well as two pathogenic fungi [*Candida albicans* 10221 (*C. albicans*), and *Aspergillus niger* (*A. niger*)]. *B. subtilis*, *S. aureus*, *E. coli*, *P. aeruginosa*, and *C. albicans* were purchased from American Type Culture Collection (ATCC), while *A. niger* clinical isolate was obtained from Assiut University Mycological Centre (AUMC). For bacterial inoculum preparation, strains were pre-cultured in Luria–Bertani broth overnight under shaking conditions at 37 °C. Then, the concentration of each strain was adjusted to achieve turbidity equivalent to 0.5 McFarland standard (1.5 × 10^8^ CFU/mL)^83^. For fungi, the cultures were streaked onto the Sabouraud Dextrose Agar (SDA) plates. Then, the plates were incubated at 25 ± 2 °C for 3–7 days. After this incubation period, the spores were harvested using a sterile scalpel blade and suspended in sterilized distilled water containing 0.01% Tween 80. The spore suspension was vortexed for 5 min to equally distributed the spores. The number of spores was counted using an improved Neubauer hemocytometer (Marienfeld, Germany), and the final concentration of the suspension was 1 × 10^6^ spores/ml.

##### Agar well diffusion method

In this experiment, one ml of microbial culture was swapped on the surface of agar plates Luria–Bertani (LB) for bacterial strains and SDA for fungi. Then, a well with a diameter of (6 mm) was punched into the agar using a sterile cork borer. After that, 100 µl of the fungal extract (5 mg/ml) was applied to each well. Gentamicin and fluconazole were used as a positive control (5 mg/ml), while DMSO 10% was a negative control.

##### Determination of minimum inhibitory (MIC), minimum bactericidal (MBC), and minimum fungicidal (MFC) concentrations of the PsEAE of the endophytic fungus *Paecilomyces* sp. (AUMC 15510)

The EtOAc crude extract from *Paecilomyces* sp. (AUMC 15510) was further assessed for its MIC, MBC, or MFC using the broth microdilution method described by Ferraro^84^. The fungal extract concentrations were prepared in 96-well microtiter plates by twofold serial dilution to get final concentrations to range from 0.06 to 1000 μg/ml. After that, each well was provided with 100 μl of culture media, 100 μl of fungal extract, and 10 μl of microbial suspension. Gentamicin and fluconazole were used as positive control, while wells containing only culture media with microbial suspension were used as a negative control. The plates were incubated at 35 ± 2 °C for 16–20 h and then scanned at 600 nm using Microplate Reader. Fungal extract's MIC was identified as the lowest concentration that completely inhibited microbial growth. To evaluate the MBC or MFC, the concentrations that showed complete inhibition of the microbial growth were streaked onto agar plates and incubated under the same conditions as previously mentioned. The complete inhibition of microbial growth on the agar surface at the lowest fungal extract concentration was defined as the MBC.

#### Antibiofilm assay

##### Qualitative detection of biofilm formation

The qualitative assessment of biofilm production by tested bacterial strains (*B. subtilis*, *S. aureus*, *E. coli*, and *P. aeruginosa*) was performed by two methods as Congo Red Agar (CRA) method and Tube Staining Method (TSM). *Staphylococcus epidermidis* ATCC 12228 was used as a non-biofilm producer reference strain. For the CRA test, the tested bacterial cultures along with the reference strain were streaked on the agar plates containing brain heart infusion broth (BHIB) (37 g/l), sucrose (50 g/l), agar (10 g/l), and Congo red dye (8 g/l) then incubating the plates at 37 °C for 48 h^85^. After incubation, the biofilm-producing bacteria grew as black colonies, while non-biofilm producers formed pink colonies. The TSM was performed according to the method described by Christensen et al.^86^ with some modifications. Briefly, 2 ml of BHIB supplemented with 5% (w/v) sucrose and 0.8% (w/v) Congo Red dye was inoculated with 200 μl of overnight culture and incubated at 37 °C for 48 h under static conditions. After that, the culture media were discarded slowly, and the tubes were washed with phosphate buffer saline (PBS pH 7.3) and dried. Further, crystal violet 2% (w/v) was used to stain the dried tubes. The tubes were washed several times with deionized water to remove the extra stain. Then, tubes were observed visually for biofilm production. The positive result of biofilm formation was considered when a visible film lined the bottom and the wall of the tube.

##### Quantitative assessment of biofilm biomass

Biofilm production by *B. subtilis*, *S. aureus*, *E. coli*, and *P. aeruginosa* was performed using the microtiter plate assay with slight modifications^87^. Briefly, a single colony from the Brain Heart Infusion Agar (BHIA) overnight bacterial culture was inoculated into BHIB supplemented with 2% glucose and incubated at 37 °C overnight in a rotary shaker at 150 rpm. Each well of 96-well flat-bottom microplate was filled with 200 μl of the bacterial suspension. Wells containing only 200 μl of cell-free media were served as a negative control. The plate was then incubated at 37 °C for 48 h. After incubation, the content of each well was carefully discarded and washed three times with 200 μl of PBS (pH 7.3) to remove the non-adherent bacterial cells. The wells containing adhered biofilm were then fixed with 200 μl of methanol for 15 min and air-dried at room temperature. Crystal violet 2% (200 μl) was used to stain the bacterial biofilm for 15 min at room temperature, and the plates were then washed three times with distilled water to remove the excess stain. Next, 200 μl of 33% glacial acetic acid was added to each well for 30 min to resolubilize the adhered biofilm. The optical density (OD) of stained biofilm at 600 nm was measured using the microtiter plate reader (BioTek EPOCH, Highland Park, Winooski, VT, USA). All biofilm experiments were performed twice in triplicate. Standard deviations and mean values of OD were calculated.

##### Biofilm inhibition assay of ethyl acetate crude extract

The antibiofilm activity of the crude extract against biofilm production by *P. aeruginosa*, *S. aureus*, and *B. subtilis* was assessed according to the method described by Yimgang et al.^88^ with some modifications. Briefly, 100 μl of overnight culture from each bacterial strain was incubated with 10 μl of crude extract at MIC, 2 MIC, and 4 MIC for 48 h at 37 °C. After this incubation period, the free-floating bacterial cells were gently removed by rinsing the wells three times with PBS (pH 7.3). Next, each well was stained with 150 μl of crystal violet 2% for 15 min. Absorbance values OD_600nm_ were measured using the microtiter plate reader (BioTek EPOCH, Highland Park, Winooski, VT, USA). Gentamicin was used as a positive control at 20 μg/ml, while wells containing only medium were considered as a negative control. The assay was done twice with three replicates. The percentage of biofilm inhibition was calculated as follows:

1 $$ \% \;{\text{of}}\;{\text{inhibition}} = \left[ {({\text{control}}\;{\text{OD}}_{{600\;{\text{nm}}}} {-}{\text{treated}}\;{\text{OD}}_{{600\;{\text{nm}}}} )/{\text{control}}\;{\text{OD}}_{{600\;{\text{nm}}}} } \right] \times 100. $$

#### Wound healing assay

Earthworms (*Lumbricus castaneus*) model was used to assess the wound healing activity of PsEAE. Earthworms have been collected from Assiut University farm and transferred to the laboratory under standard conditions for the experiment (25–28 °C with a 12 h day:12 h night). Worms are stored in plastic packing containers with wet soil, and dried cattle manure is delivered to the soil for the worms' feed. The wound was made by using a sterile scalpel. A preliminary study was conducted for different concentrations of the extract 5, 10, 15, 20, 25, 30 mg and mixed in an equal amount of Vaseline to be applied topically to the wounds. The worms that received 5, 10, 15 mg of PsEAE showed the most enhanced wound healing among all the groups. Therefore, the worms were divided randomly into five groups (n = 5) as follows:

Group 1 (control): the control group is not subjected to any injury.

Group 2 (untreated): This group was subjected to injury and received Vaseline only.

Group 3: The wounds in the worms were treated with 5 mg of PsEAE mixed with Vaseline.

Group 4: The wounds in the worms were treated with 10 mg of PsEAE mixed with Vaseline.

Group 5: The wounds in the worms were treated with 15 mg of PsEAE mixed with Vaseline.

PsEAE was applied trice every day for 6 days, according to Abd Ellah et al.^26^. The worms were maintained in a Petri dish containing wetted filter paper to achieve the required humidity level. Wound diameter was assessed after every day and quantified in millimeters.

##### Histological investigations

Earthworm tissue longitudinal sections (5 μm from different groups were mounted on slides and dried overnight at 37 °C, de-waxed in xylene and hydrated in a graded series of alcohols, and hematoxylin and eosin were used for staining^89^.

##### SEM analysis

The worms from different groups were fixed in 5% glutaraldehyde in sodium cacodylate buffer for 1.5 h, rinsed in distilled water, and dehydrated in ethanol, followed by drying. Then, samples were mounted on stubs, coated with carbon or gold then examined by Joel JSM 35 SEM at 20 kv.

##### Semithin sections samples preparation

Getting ready for semithin portions, the earthworms were fixated in 4% cold glutaraldehyde, washed up to four times in phosphate buffer (pH 7.2), followed by fixation in 1% osmium tetroxide (OsO4) for 2 h, and rinsed four times in the same buffer. The concentration of ethyl alcohol was gradually increased to achieve dehydration. To remove alcohol residues, tissue specimens were soaked in propylene oxide for 30 min, then in the mixture of (1:1, v/v) of propylene oxide plus Epon 812 for another 30 min followed by soaking in Epon 812 for 4 h. The tissue blocks were inserted into capsules, together with the embedding mixture, and then polymerized in a 60 °C oven for two days. Parts of the LKB ultramicrotome were sliced semithin of 0.5-μm thickness that were subjected to toluidine blue staining^90^.

##### TEM analysis

Tissue localization was evaluated on semithin components, and ultrathin parts were created as needed. Leica AG ultramicrotome was used to cut ultrathin pieces (50–80 nm) that were stained with uranyl acetate and lead citrate. TEM (JEOL, 100 CXII) at 80 kV was used to earthworm parts from different groups. Electron micrographs were acquired, reconstructed, and evaluated to study the selected semithin regions using Photoshop software.

## Supplementary Information

Supplementary Figures.

## Data Availability

All data generated or analysed during this study are included in this published article. The *Paecilomyces* sp. strain in this study was preserved as frozen and lyophilized cultures and added to the culture collections of the Assiut University Mycological Centre (AUMC) as AUMC 15510 (Stem of *Cornulaca monacantha*, Wadi El-Assiuti Protectorate, Assiut Governorate, Egypt). ITS and LSU sequences of the strain were uploaded to GenBank database as OP429630 and ON685324, respectively (https://www.ncbi.nlm.nih.gov/genbank).

## References

[1] Organization, W. H. World Health Organization annual report 2019 WHO Country Office Lebanon: Health for all. (2020).

[2] Brown ED, Wright GD (2016). Antibacterial drug discovery in the resistance era. Nature.

[3] Nikaido H (2009). Multidrug resistance in bacteria. Annu. Rev. Biochem..

[4] Mangoni ML, McDermott AM, Zasloff M (2016). Antimicrobial peptides and wound healing: Biological and therapeutic considerations. Exp. Dermatol..

[5] Savitskaya I, Shokatayeva D, Kistaubayeva A, Ignatova L, Digel I (2019). Antimicrobial and wound healing properties of a bacterial cellulose based material containing *B. subtilis* cells. Heliyon.

[6] Enyedi B, Niethammer P (2015). Mechanisms of epithelial wound detection. Trends Cell Biol..

[7] Lau K, Paus R, Tiede S, Day P, Bayat A (2009). Exploring the role of stem cells in cutaneous wound healing. Exp. Dermatol..

[8] Hu MS (2014). Tissue engineering and regenerative repair in wound healing. Ann. Biomed. Eng..

[9] Ramot Y (2015). The role of PPAR γ-mediated signalling in skin biology and pathology: New targets and opportunities for clinical dermatology. Exp. Dermatol..

[10] Guo SA, Di Pietro LA (2010). Factors affecting wound healing. J. Dental Res..

[11] Mustoe T (2004). Understanding chronic wounds: A unifying hypothesis on their pathogenesis and implications for therapy. Am. J. Surg..

[12] Demidova-Rice TN, Hamblin MR, Herman IM (2012). Acute and impaired wound healing: pathophysiology and current methods for drug delivery, part 1: Normal and chronic wounds: biology, causes, and approaches to care. Adv. Skin Wound Care.

[13] Gallo RL, Hooper LV (2012). Epithelial antimicrobial defence of the skin and intestine. Nat. Rev. Immunol..

[14] Mancl KA, Kirsner RS, Ajdic D (2013). Wound biofilms: Lessons learned from oral biofilms. Wound Repair Regeneration.

[15] Wolcott R (2010). Chronic wounds and the medical biofilm paradigm. J. Wound Care.

[16] Rajpaul K (2015). Biofilm in wound care. Br. J. Community Nurs..

[17] Ovington L (2003). Bacterial toxins and wound healing. Ostomy Wound Manage..

[18] Ruddaraju LK, Pammi SVN, Sankar Guntuku G, Padavala VS, Kolapalli VRM (2020). A review on anti-bacterials to combat resistance: From ancient era of plants and metals to present and future perspectives of green nano technological combinations. Asian J. Pharm. Sci..

[19] Rambold G, Stadler M, Begerow D (2013). Mycology should be recognized as a field in biology at eye level with other major disciplines–a memorandum. Mycol. Prog..

[20] Gupta S, Chaturvedi P, Kulkarni MG, Van Staden J (2020). A critical review on exploiting the pharmaceutical potential of plant endophytic fungi. Biotechnol. Adv..

[21] Helaly SE, Thongbai B, Stadler M (2018). Diversity of biologically active secondary metabolites from endophytic and saprotrophic fungi of the ascomycete order Xylariales. Nat. Prod. Rep..

[22] Sandargo B (2019). Biological and chemical diversity go hand in hand: Basidiomycota as source of new pharmaceuticals and agrochemicals. Biotechnol. Adv..

[23] Salem SH (2022). GC–MS analysis, cytotoxicity, and molecular docking studies of bioactive alkaloids extracted from tomato leaves inoculated with endophytic fungus Beauveria sp. AUMC 15401. J. Food Process. Preserv..

[24] Dai ZB, Wang X, Li GH (2020). Secondary metabolites and their bioactivities produced by paecilomyces. Molecules.

[25] Abd El-Aziz FE-ZA, Hetta HF, Abdelhamid BN, Abd Ellah NH (2022). Antibacterial and wound-healing potential of PLGA/spidroin nanoparticles: A study on earthworms as a human skin model. Nanomedicine.

[26] Abd Ellah NH, Abd El-Aziz FEZA, Abouelmagd SA, Abd El-Hamid BN, Hetta HF (2019). Spidroin in carbopol-based gel promotes wound healing in earthworm's skin model. Drug Develop. Res..

[27] El-Aziz A, Ali MF (2021). Towards study of UV-C radiation effect on earthworms and isopods via electron microscopy. Egypt. Acad. J. Biol. Sci. B. Zool..

[28] Bernardi APM (2005). Benzophenones from Hypericum c arinatum. J. Nat. Prod..

[29] Mantovani G, Fukushima WY, Cho AB, Aita MA, Mazzetti MV (2009). Use of earthworms for microsurgery training. J. Reconstr. Microsurg..

[30] Albro PW, Bilski P, Corbett JT, Schroeder JL, Chignell CF (1997). Photochemical reactions and phototoxicity of sterols: Novel self-perpetuating mechanism for lipid photooxidation. Photochem. Photobiol..

[31] Misra R, Lal K, Farooq M, Hans R (2005). Effect of solar UV radiation on earthworm (Metaphire posthuma). Ecotoxicol. Environ. Saf..

[32] Wu Y-Z (2018). Bysspectin A, an unusual octaketide dimer and the precursor derivatives from the endophytic fungus Byssochlamys spectabilis IMM0002 and their biological activities. Eur. J. Med. Chem..

[33] Abd El-Rahman TM, Tharwat NA, Abo El-Souad SM, El-Beih AA, El-Diwany AI (2020). Biological activities and variation of symbiotic fungi isolated from Coral reefs collected from Red Sea in Egypt. Mycology.

[34] Amer M, Barakat K, Hassanein A (2019). Phthalate derivatives from marine Penicillium decumbens and its synergetic effect against sepsis bacteria. Biointerface Res. Appl. Chem.

[35] Roy RN (2020). Bioactive natural derivatives of phthalate ester. Crit. Rev. Biotechnol..

[36] Huang L (2021). Phthalic acid esters: Natural sources and biological activities. Toxins.

[37] Holt G (2017). Shigatoxin encoding Bacteriophage ϕ24B modulates bacterial metabolism to raise antimicrobial tolerance. Sci. Rep..

[38] Cardoso CRB, Souza MA, Ferro EAV, Favoreto S, Pena JDO (2004). Influence of topical administration of n-3 and n-6 essential and n-9 nonessential fatty acids on the healing of cutaneous wounds. Wound Repair Regeneration.

[39] Feng X, Cheng G, Chen S-Y, Yang H, Huang W (2010). Evaluation of the burn healing properties of oil extraction from housefly larva in mice. J. Ethnopharmacol..

[40] Kanetsuna F (1985). Bactericidal effect of fatty acids on mycobacteria, with particular reference to the suggested mechanism of intracellular killing. Microbiol. Immunol..

[41] Bailey A, De Lucca A, Moreau J (1989). Antimicrobial properties of some erucic acid-glycolic acid derivatives. J. Am. Oil Chem. Soc..

[42] Kim Y-G (2018). Herring oil and omega fatty acids inhibit Staphylococcus aureus biofilm formation and virulence. Front. Microbiol..

[43] Renda G (2013). Comparative assessment of dermal wound healing potentials of various Trifolium L. extracts and determination of their isoflavone contents as potential active ingredients. J. Ethnopharmacol..

[44] Tang Z (2020). Isolation and identification of flavonoid-producing endophytic fungi from medicinal plant Conyza blinii H. Lév that exhibit higher antioxidant and antibacterial activities. PeerJ.

[45] Alshehri, M. M. *et al.* Therapeutic potential of isoflavones with an emphasis on daidzein. *Oxidat. Med. Cell. Longevity*. **2021** (2021). 10.1155/2021/6331630 PMC8448605 34539970

[46] Mssillou I (2022). Investigation on wound healing effect of Mediterranean medicinal plants and some related phenolic compounds: A review. J. Ethnopharmacol..

[47] Garcia Forero A (2019). Photoprotective and antigenotoxic effects of the flavonoids apigenin, naringenin and pinocembrin. Photochem. Photobiol..

[48] Salehi B (2019). The therapeutic potential of naringenin: a review of clinical trials. Pharmaceuticals.

[49] Soberón JR (2020). Antifungal activity and toxicity studies of flavanones isolated from Tessaria dodoneifolia aerial parts. Heliyon.

[50] Genaro-Mattos TC, Maurício ÂQ, Rettori D, Alonso A, Hermes-Lima M (2015). Antioxidant activity of caffeic acid against iron-induced free radical generation—A chemical approach. PLoS ONE.

[51] Magnani C, Isaac VLB, Correa MA, Salgado HRN (2014). Caffeic acid: A review of its potential use in medications and cosmetics. Anal. Methods.

[52] Romana-Souza B, Dos Santos JS, Monte-Alto-Costa A (2018). Caffeic acid phenethyl ester promotes wound healing of mice pressure ulcers affecting NF-κB, NOS2 and NRF2 expression. Life Sci..

[53] David AVA, Arulmoli R, Parasuraman S (2016). Overviews of biological importance of quercetin: A bioactive flavonoid. Pharmacogn. Rev..

[54] Yang, D., Wang, T., Long, M. & Li, P. Quercetin: its main pharmacological activity and potential application in clinical medicine. *Oxidat. Med. Cell. Longevity*. **2020** (2020). 10.1155/2020/8825387 PMC7790550 33488935

[55] Osonga FJ (2019). Antimicrobial activity of a new class of phosphorylated and modified flavonoids. ACS Omega.

[56] Mi Y (2022). Quercetin promotes cutaneous wound healing in mice through Wnt/β-catenin signaling pathway. J. Ethnopharmacol..

[57] Mou Y (2013). Antimicrobial and antioxidant activities and effect of 1-hexadecene addition on palmarumycin C2 and C3 yields in liquid culture of endophytic fungus Berkleasmium sp. Dzf12. Molecules.

[58] Marrufo T (2013). Chemical composition and biological activity of the essential oil from leaves of Moringa oleifera Lam. cultivated in Mozambique. Molecules.

[59] Chatterjee S, Karmakar A, Azmi SA, Barik A (2018). Proceedings of the Zoological Society.

[60] Chandrasekaran M, Senthilkumar A, Venkatesalu V (2011). Antibacterial and antifungal efficacy of fatty acid methyl esters from the leaves of Sesuvium portulacastrum L. Eur. Rev. Med. Pharmacol. Sci..

[61] Hema R, Kumaravel S, Alagusundaram K (2011). GC/MS determination of bioactive components of Murraya koenigii. J. Am. Sci..

[62] Shaaban MT, Ghaly MF, Fahmi SM (2021). Antibacterial activities of hexadecanoic acid methyl ester and green-synthesized silver nanoparticles against multidrug-resistant bacteria. J. Basic Microbiol..

[63] Pereira LM (2008). Effect of oleic and linoleic acids on the inflammatory phase of wound healing in rats. Cell Biochem. Function.

[64] Magdalon J (2012). Oral administration of oleic or linoleic acids modulates the production of inflammatory mediators by rat macrophages. Lipids.

[65] Hamazaki K (2016). Is vaccenic acid (18: 1t n-7) associated with an increased incidence of hip fracture? An explanation for the calcium paradox. Prostaglandins Leukot. Essent. Fatty Acids.

[66] Gevorgyan S (2022). Structural characterization and antibacterial activity of silver nanoparticles synthesized using a low-molecular-weight Royal Jelly extract. Sci. Rep..

[67] Pankey GA, Sabath L (2004). Clinical relevance of bacteriostatic versus bactericidal mechanisms of action in the treatment of Gram-positive bacterial infections. Clin. Infect. Dis..

[68] Stepanović S (2007). Quantification of biofilm in microtiter plates: overview of testing conditions and practical recommendations for assessment of biofilm production by staphylococci. APMIS.

[69] Pletzer D, Hancock RE (2016). Antibiofilm peptides: Potential as broad-spectrum agents. J. Bacteriol..

[70] Cheng Y, Qin J, Huang Y, Wang T (2022). The antimicrobial effects of PLGA microspheres containing the antimicrobial peptide OP-145 on clinically isolated pathogens in bone infections. Sci. Rep..

[71] Jhonson SC (2012). Autofluorescence in BrdU-positive cells and augmentation of regeneration kinetics by riboflavin. Stem Cells Develop..

[72] Hulikere MM, Joshi CG, Ananda D, Poyya J, Nivya T (2016). Antiangiogenic, wound healing and antioxidant activity of Cladosporium cladosporioides (Endophytic Fungus) isolated from seaweed (Sargassum wightii). Mycology.

[73] Abdel-Motaal FF (2022). Comparative studies on the antioxidant, antifungal, and wound healing activities of Solenostemma arghel ethyl acetate and methanolic extracts. Appl. Sci..

[74] Ibrahim N, Abbas H, El-Sayed NS, Gad HA (2022). Rosmarinus officinalis L. hexane extract: Phytochemical analysis, nanoencapsulation, and in silico, in vitro, and in vivo anti-photoaging potential evaluation. Sci. Rep..

[75] Cinar S, Hatipoglu R, Gundel FD, Aktas A, Mustafa A (2014). Performances of some perennial warm season grasses alfalfa (Medicago sativa L.) mixtures under Mediterranean conditions. Turk. J. Field Crops.

[76] Mizutani K, Ogawa H, Saito J, Oka K (2002). Fictive locomotion induced by octopamine in the earthworm. J. Exp. Biol..

[77] Moisenovich M (2015). Doklady Biochemistry and Biophysics.

[78] Araújo WL (2002). Diversity of endophytic bacterial populations and their interaction with Xylella fastidiosa in citrus plants. Appl. Environ. Microbiol..

[79] White TJ, Bruns T, Lee S, Taylor J (1990). Amplification and direct sequencing of fungal ribosomal RNA genes for phylogenetics. PCR Protocols.

[80] Rehner SA, Samuels GJ (1994). Taxonomy and phylogeny of Gliocladium analysed from nuclear large subunit ribosomal DNA sequences. Mycol. Res..

[81] Kumar S, Stecher G, Li M, Knyaz C, Tamura K (2018). MEGA X: molecular evolutionary genetics analysis across computing platforms. Mol. Biol. Evol..

[82] Rom, W. R. o. M. S. D. N. S. D. C. (Wiley, 1998).

[83] Bhalodia NR, Shukla V (2011). Antibacterial and antifungal activities from leaf extracts of Cassia fistula l.: An ethnomedicinal plant. J. Adv. Pharm. Technol. Res..

[84] Ferraro, M. J. *Methods for dilution antimicrobial susceptibility tests for bacteria that grow aerobically*. (NCCLS, 2000). 10.1128/aac.44.6.1694-1696.2000 PMC89935 10817731

[85] Knobloch JK-M, Horstkotte MA, Rohde H, Mack D (2002). Evaluation of different detection methods of biofilm formation in Staphylococcus aureus. Med. Microbiol. Immunol..

[86] Christensen GD, Simpson WA, Bisno AL, Beachey EH (1982). Adherence of slime-producing strains of Staphylococcus epidermidis to smooth surfaces. Infect. Immun..

[87] Cruz CD, Shah S, Tammela P (2018). Defining conditions for biofilm inhibition and eradication assays for Gram-positive clinical reference strains. BMC Microbiol..

[88] Yimgang LV, Kouipou Toghueo RM, Mbekou IMK, Dize D, Boyom FF (2022). Crude metabolites from endophytic fungi inhabiting Cameroonian Annona muricata inhibit the causative agents of urinary tract infections. PLoS ONE.

[89] Corrin B (1981). Carleton's histological technique. J. Clin. Pathol..

[90] Gupta P (1983). Ultrastructural study on semithin section. Sci Tools.

