# Supplementary material for: The antimicrobial, antibiofilm, and wound healing properties of ethyl acetate crude extract of an endophytic fungus Paecilomyces sp. (AUMC 15510) in earthworm model
Source: Sci Rep. 2022 Nov 10;12:19239. doi: 10.1038/s41598-022-23831-4 (PMC9649741; doi:10.1038/s41598-022-23831-4)
Supplement: Supplementary file 1 — Supplementary Figures. [file 41598_2022_23831_MOESM1_ESM.pdf]

The antimicrobial, antibiofilm, and wound healing properties of ethyl acetate crude extract of an endophytic fungus *Paecilomyces* sp. (AUMC 15510) in earthworm model

Authors and affiliations:

Shimaa H. Salem[1], Saad S. El-Maraghy[1], Ahmed Y. Abdel-Mallek[2], Mohamed A. A. Abdel-Rahman[3], Emad H.M. Hassanein[4], Osama A. Al-Bedak [5], Fatma El-Zahraa A Abd El-Aziz[6*]

[1] Fungal Physiology Laboratory, Botany and Microbiology Department, Faculty of Science, Assiut University, Assiut, Egypt.

[2] Mycology Laboratory, Botany and Microbiology Department, Faculty of Science, Assiut University, Assiut, Egypt.

[3] Plant Protection Research Institute, Agricultural Research Center, Dokki, Giza, Egypt

[4] Department of Pharmacology & Toxicology, Faculty of Pharmacy, Al-Azhar University-Assiut Branch, Assiut, Egypt.

[5] Assiut University Mycological Centre (AUMC), Assiut University, Assiut, Egypt.

[6] Department of Zoology, Faculty of Science, Assiut University, Assiut, Egypt.

[*] Corresponding author at Department of Zoology, Faculty of Science, Assiut University,

Assiut, Egypt 71516. Tel: +201125853555

E-mail: Fatma.abdelgalil1@science.aun.edu.eg (Fatma El-Zahraa A Abd El-Aziz, PhD).

ORCID ID: 0000-0001-7260-7598.

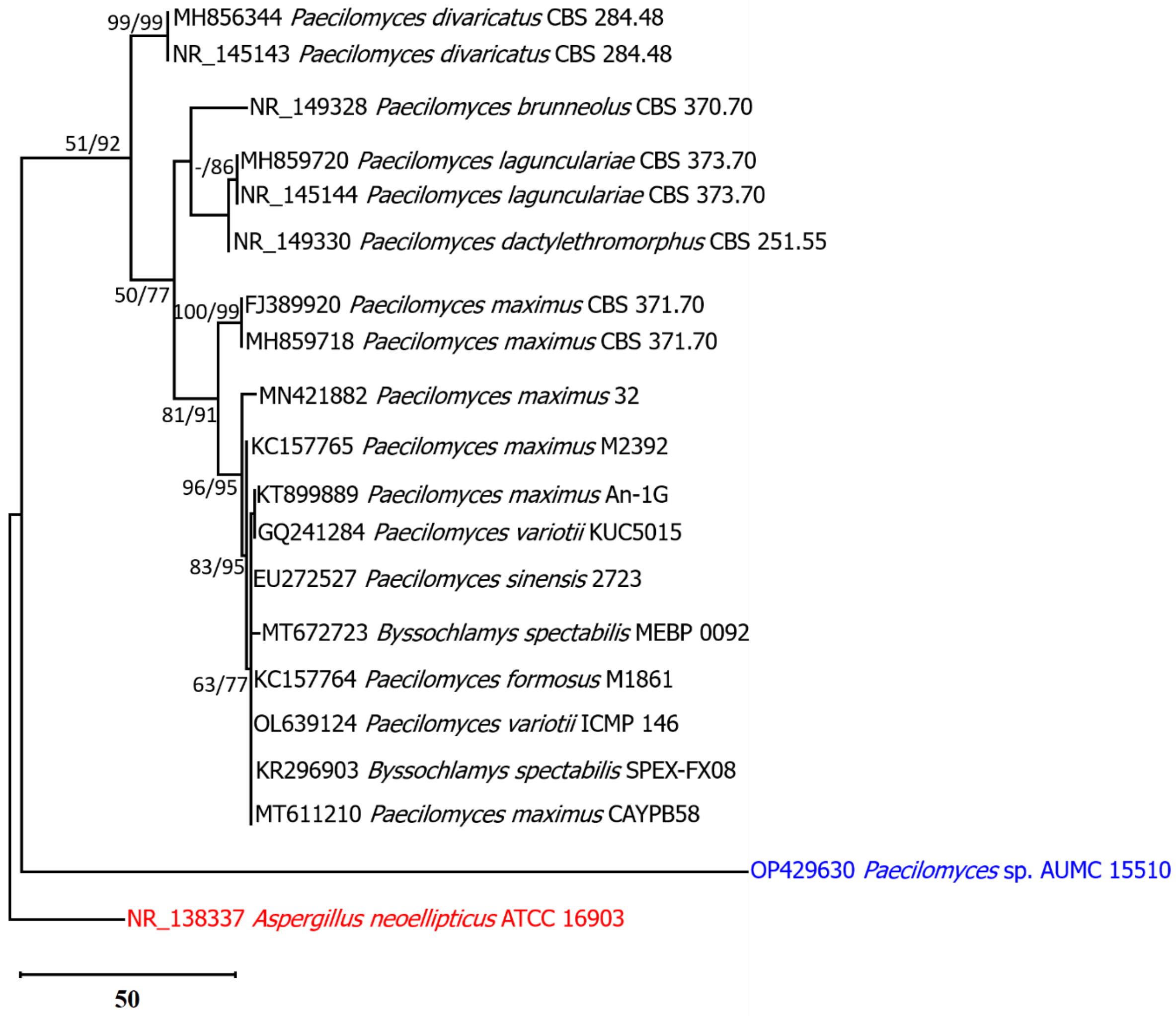

Figure S1. Phylogenetic analyses of endophytic fungus *Paecilomyces* sp. (AUMC 15510) isolated from *C. monacantha*. *Aspergillus neoellipticus* ATCC 16903 was designated as an outgroup taxon.

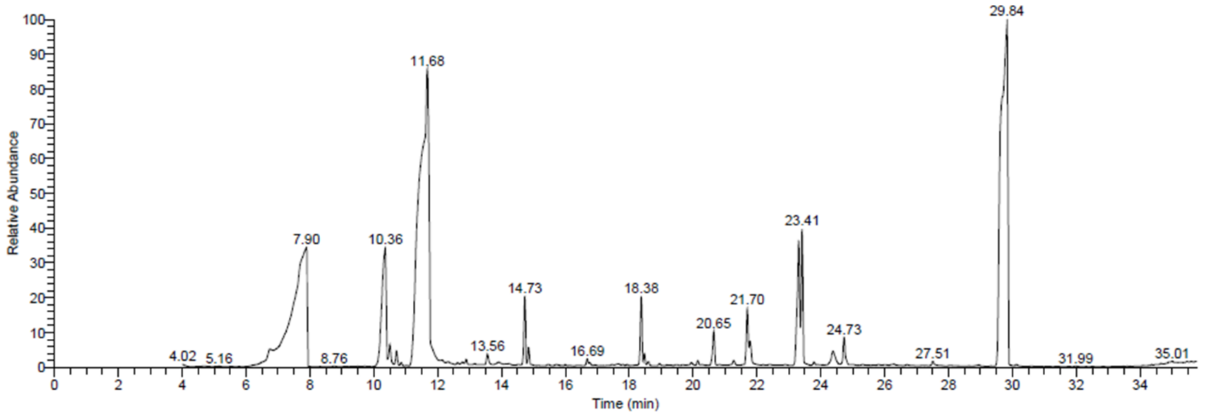

Figure S2. GC-MS chromatogram of ethyl acetate crude extract of *Paecilomyces* sp. (AUMC-15510)

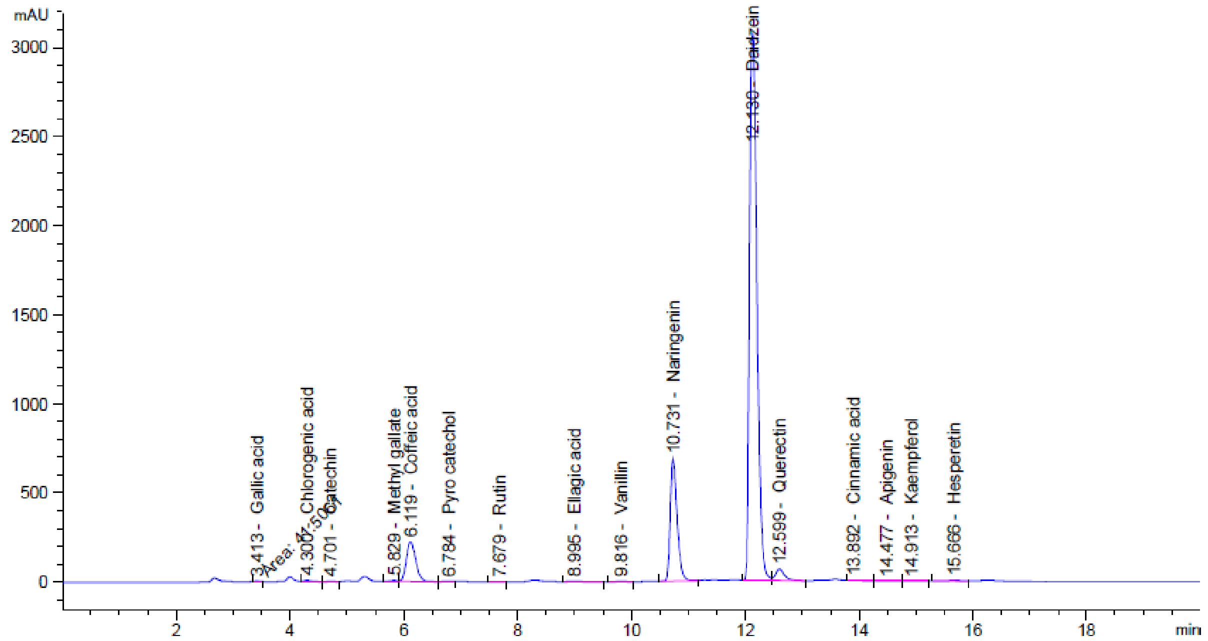

Figure S3. HPLC chromatogram of ethyl acetate crude extract of *Paecilomyces* sp. (AUMC-15510)

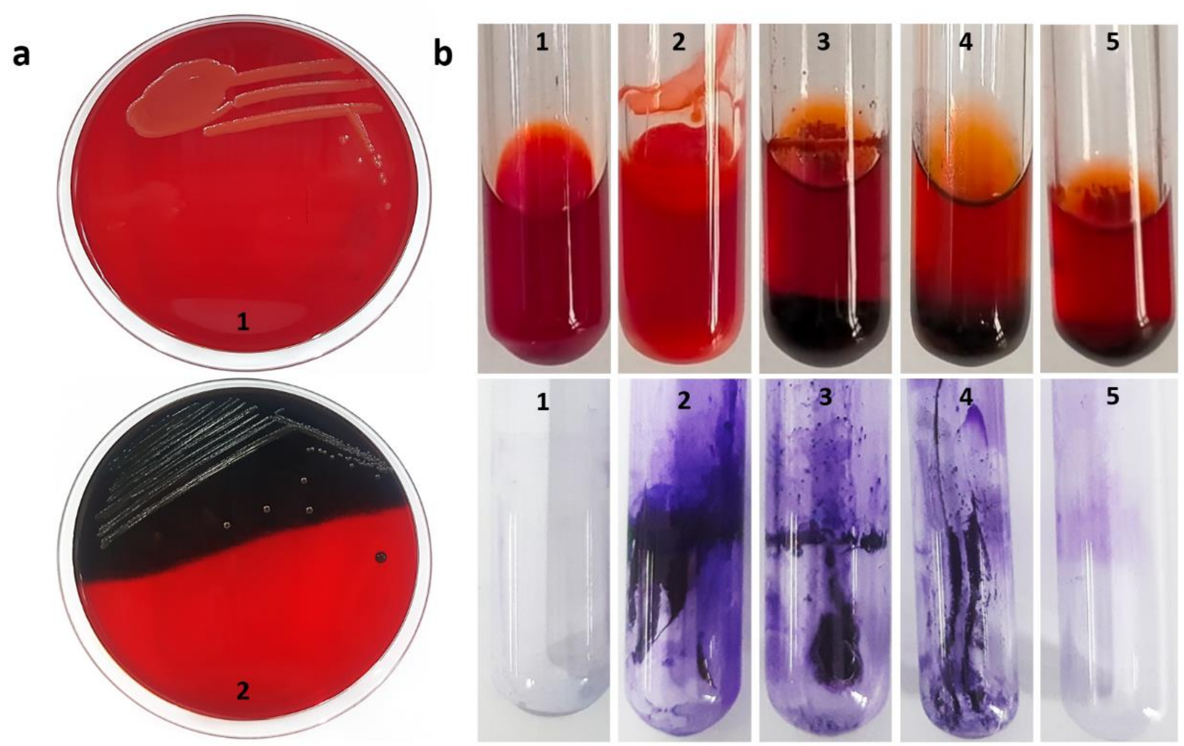

Figure S4. (**a**) Biofilm formation on Congo Red Agar medium. Plate 1: non-biofilm producer reference strain *S. epidermidis* ATCC 12228, Plate 2: biofilm producer strain with black colonies. (**b**) Biofilm production by tube staining method. Tube 1: control (Broth medium only), Tube 2: biofilm formation by *P. aeruginosa*, Tube 3: *S. aureus* biofilm, Tube 4: *B. subtilis* biofilm, Tube 5: *E. coli* biofilm.

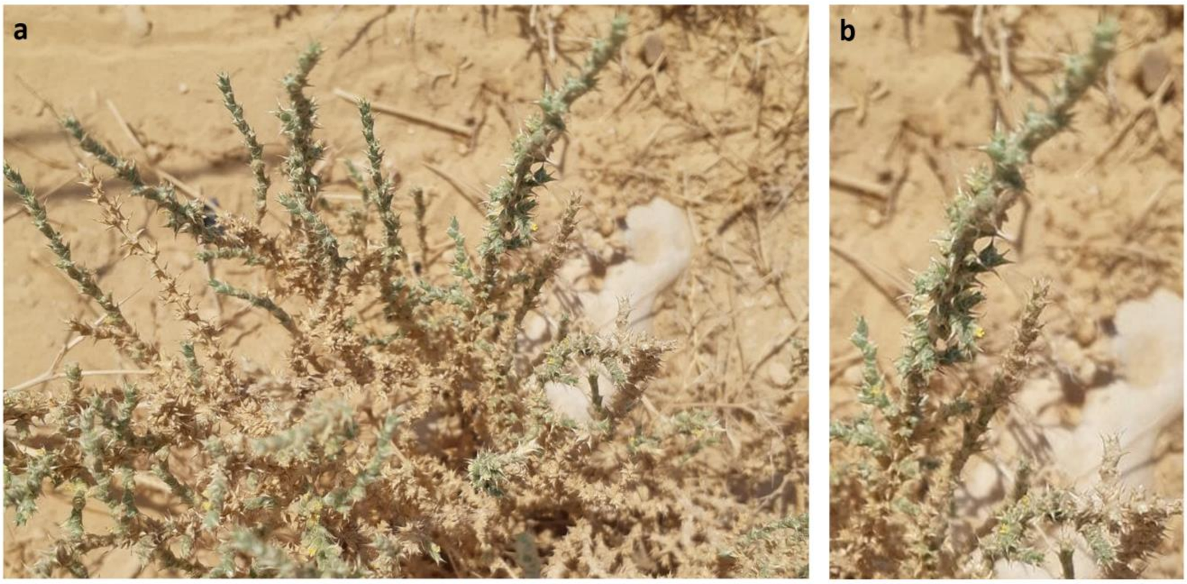

Figure S5. *Cornulaca monacantha*: (**a**) woody shrub (**b**) *Cornulaca* stem bearing green scale-like leaves with a single spine at the tip.
